# ROS-dependent activation of RhoA/Rho-kinase in pulmonary artery: Role of Src-family kinases and ARHGEF1

**DOI:** 10.1016/j.freeradbiomed.2017.06.022

**Published:** 2017-09

**Authors:** Charles E. MacKay, Yasin Shaifta, Vladimir V. Snetkov, Asvi A. Francois, Jeremy P.T. Ward, Greg A. Knock

**Affiliations:** a Asthma, Allergy & Lung Biology, Faculty of Life Sciences & Medicine, King's College London, London, United Kingdom; b Cardiovascular Division, Faculty of Life Sciences & Medicine, King's College London, London, United Kingdom

**Keywords:** Vascular smooth muscle, Tyrosine kinases, Src-family kinases, Reactive oxygen species, Hypoxia, Rho-kinase, Guanine nucleotide exchange factors, Pulmonary artery

## Abstract

The role of reactive oxygen species (ROS) in smooth muscle contraction is poorly understood. We hypothesised that G-protein coupled receptor (GPCR) activation and hypoxia induce Rho-kinase activity and contraction in rat intra-pulmonary artery (IPA) via stimulation of ROS production and subsequent Src-family kinase (SrcFK) activation.

The T-type prostanoid receptor agonist U46619 induced ROS production in pulmonary artery smooth muscle cells (PASMC). U46619 also induced c-Src cysteine oxidation, SrcFK auto-phosphorylation, MYPT-1 and MLC_20_ phosphorylation and contraction in IPA, and all these responses were inhibited by antioxidants (ebselen, Tempol). Contraction and SrcFK/MYPT-1/MLC_20_ phosphorylations were also inhibited by combined superoxide dismutase and catalase, or by the SrcFK antagonist PP2, while contraction and MYPT-1/MLC_20_ phosphorylations were inhibited by the Rho guanine nucleotide exchange factor (RhoGEF) inhibitor Y16. H_2_O_2_ and the superoxide-generating quinoledione LY83583 both induced c-Src oxidation, SrcFK auto-phosphorylation and contraction in IPA. LY83583 and H_2_O_2_-induced contractions were inhibited by PP2, while LY83583-induced contraction was also inhibited by antioxidants and Y16. SrcFK auto-phosphorylation and MYPT-1/MLC_20_ phosphorylation was also induced by hypoxia in IPA and this was blocked by mitochondrial inhibitors rotenone and myxothiazol. In live PASMC, sub-cellular translocation of RhoA and the RhoGEF ARHGEF1 was triggered by both U46619 and LY83583 and this translocation was blocked by antioxidants and PP2. RhoA translocation was also inhibited by an ARHGEF1 siRNA. U46619 enhanced ROS-dependent co-immunoprecipitation of ARHGEF1 with c-Src.

Our results demonstrate a link between GPCR-induced cytosolic ROS or hypoxia-induced mitochondrial ROS and SrcFK activity, Rho-kinase activity and contraction. ROS and SrcFK activate RhoA via ARHGEF1.

## Highlights

• GPCR-induced contraction of intra-pulmonary artery (IPA) is ROS-dependent.
• Endogenous and exogenous ROS induce RhoA translocation and Rho-kinase activity in IPA.
• Endogenous and exogenous ROS also activate Src-family kinases (SrcFK) in IPA.
• SrcFK and ARHGEF1 are required for ROS-induced RhoA and Rho-kinase activity.
• SrcFK may induce ROS-dependent activation of RhoA via ARHGEF1.

## Graphical abstract

**Figure:**
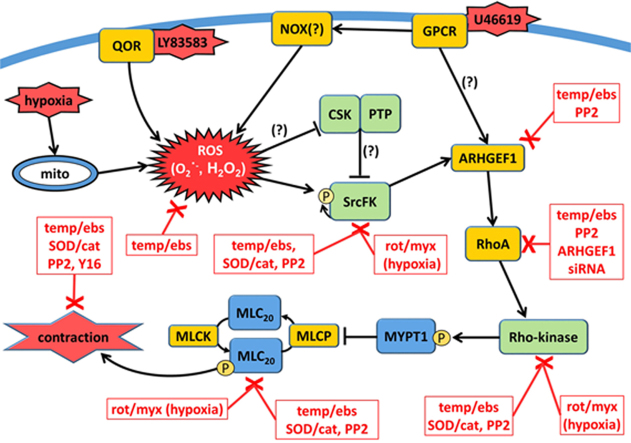

## 1 Introduction

Reactive oxygen species (ROS), previously thought to occur solely as damaging bi-products of metabolism, are now recognised as bona fide second messengers in normal cellular function, being produced in response to multiple stimuli, including G-protein-coupled receptor (GPCR) agonists and hypoxia [1], [2], presumably exerting their effects through reversible oxidation of specific target proteins, primarily on cysteine residues [3], [4], [5]. Normally, the production and degradation of ROS is tightly controlled by cytosolic or membrane-bound oxidoreductase enzymes, antioxidant enzymes and cellular redox buffers [6], [7], but in cardiovascular disease this control is lost, resulting in oxidative stress [8]. GPCR induce vascular ROS production primarily via NADPH oxidase [1], [7], while in intra-pulmonary arteries (IPA), hypoxia does so via the mitochondrial electron transport chain (complex III), an essential step in hypoxic pulmonary vasoconstriction [2], [9], [10]. Most contractile stimuli, including GPCR and hypoxia, exert their effects on smooth muscle cross-bridge cycling via a combination of raised [Ca^2+^]_i_ and inhibition of myosin light-chain phosphatase [11], [12], [13], the latter being dependent on activation of Rho-kinase [14]. Applied exogenously, both superoxide (O_2_^**∙**-^) and its dismutation product H_2_O_2_ have contractile effects in pulmonary artery, but previous work from our laboratory suggests that exogenous H_2_O_2_ *primarily* does so via activation of PKC and elevation of [Ca^2+^]_i_, while artificially induced cytosolic superoxide *primarily* activates Rho-kinase [15], [16].

The role of endogenous ROS in Rho-kinase activation by GPCR or hypoxia remains to be fully characterised because the signalling pathway(s) through which ROS may activate Rho-kinase are unclear. Regardless of the initial stimulus, Rho-kinase activity usually requires prior activation of monomeric G-proteins of the Rho family (including RhoA), which in turn normally requires prior activation of guanine nucleotide exchange factors selective for the Rho family of G-proteins (RhoGEFs) [17]. Thus, either RhoA itself is directly ROS-sensitive [18] or RhoGEF activation may be triggered by ROS independently of the canonical G_12_/_13_ interaction [19], [20].

Increased Rho-kinase activity contributes to acute hypoxic pulmonary vasoconstriction [11], [12], as well as elevated pulmonary artery pressure in chronic hypoxia-induced pulmonary hypertension [21], but how this increased activity is mediated remains unclear. Non-receptor tyrosine kinases contribute to aberrant migratory and proliferative responses in oxidative stress-induced vascular remodelling [22], [23], but are also involved in normal contractile function in vascular smooth muscle. For example, we showed that in IPA both GPCR-induced and hypoxia-induced Rho-kinase activity and contraction was dependent on prior activation of Src-family kinases (SrcFK) [11], [24]. In non-muscle cells, c-Src has been shown to be directly ROS-sensitive, with cysteine oxidation enhancing its activity [25], though it may also be activated indirectly through oxidative inhibition of its negative regulators, such as c-Src kinase or specific tyrosine phosphatases (reviewed in [4], [25]). It has not been determined whether SrcFK are ROS-sensitive in IPA, nor whether ROS- and/or SrcFK-mediated activation of RhoA/Rho-kinase occurs as part of the same signalling pathway in response to GPCR or hypoxia, but it is possible that SrcFK act as intermediaries between ROS and RhoA/Rho-kinase. We therefore hypothesised that SrcFK act as key mediators of ROS signalling in IPA, contributing to GPCR and hypoxia-induced RhoA/Rho-kinase activity and contraction. We also examined the role of ARHGEF1, an RGS-domain containing RhoGEF that has previously been shown to be activated by tyrosine phosphorylation [26].

## 2 Methods

### 2.1 Tissue and cell culture

This study conforms with UK Home Office regulations and Directive 2010/63/EU of the European Parliament. Adult male Wistar rats were killed by lethal overdose of pentobarbital (~50 mg/kg i.p.). The lungs were excised and placed in cold physiological saline solution (PSS, composition in mM: 118NaCl, 24 NaHCO_3,_ 1 MgSO_4_, 4 KCL, 5.56 D-glucose, 0.434 NaH_2_PO_4_, 1.8 CaCl_2_, pH 7.4). Small intra-pulmonary arteries (IPA; 200–500 µm i.d.) were dissected free of surrounding parenchyma and either used for protein extraction and immunoblotting, mounted on a myograph for measurement of contractile force, or used for preparation of cultured pulmonary artery smooth muscle cells (PASMC). PASMC were dispersed by enzymatic digestion (collagenase type XI, papain, trypsin inhibitor), grown to passage 3–4 in DMEM and serum-starved for 24 h prior to use. Each batch of cells was verified as smooth muscle by immunostaining for smooth muscle α-actin, calponin and desmin, as shown previously [1].

### 2.2 Contractile force measurement

IPA rings were mounted on a Mulvany-Halpern wire myograph (DMT.dk) bathed in PSS and gassed with 95% air/5% CO_2_ (pH 7.4) at 37 °C. Vessels were stretched and pre-conditioned by stimulation with repeated exposures to 80 mmol/l K^+^ PSS (KPSS, equimolar substitution for NaCl), with resting tone being set at 1–3 mN, as previously described [24]. Experiments were performed after ~30 min to allow for stabilization. Tension was recorded using Acquisition Engine software (Cairn Research Ltd, Faversham, UK).

### 2.3 Immuno-precipitation (IP) and Western blot (WB)

Following isolation and treatment with U46619 under normoxic conditions (5% CO_2_/95% air) or hypoxic conditions (1% O_2_/5% CO_2_, 94% N_2_), IPA tissue samples were snap frozen in liquid nitrogen, homogenised and protein extracted for IP and/or SDS-PAGE and Western blot. For IP followed by WB, protein was extracted in TRIS-buffered saline with triton (1%), whereas for immediate WB, protein was extracted in TRIS-buffered saline with SDS (5%). IP was performed using the Pierce™ Crosslink Magnetic IP/Co-IP Kit (8805) according to the manufacturer instructions. Briefly, lysates were incubated with anti-c-Src antibody (1:20, Cell Signalling) and then covalently bound to protein A/G Magnetic Beads (Pierce) overnight at 4 °C. Following appropriate washing steps and elution, immuno-precipitates were re-suspended in TRIS-SDS sample buffer.

For SDS-PAGE, gel total protein loading was normalised by BCA assay. Membranes were blocked for 1hr at room temperature with 5% skimmed milk in TBS-buffered saline with 0.05% Tween (TBS-T) then probed with primary antibodies overnight at 4 °C (in 5% milk/TBS-T). Dilutions were optimised for each primary antibody, typically 1:1000, followed by horseradish peroxidase-conjugated secondary antibodies for 1hr at room temperature (1:3000 in 5% milk/TBS-T). Membranes were first probed with anti-phospho-antibodies, then stripped for 1hr (Pierce stripping buffer), re-blocked and re-probed with anti-total antibodies. Protein bands were visualised using either SuperSignal West Femto chemiluminescent substrate (Thermo Scientific) or ECL Prime (Amersham, GE heatlthcare) in a Biorad ChemiDoc XRS+ Gel Imaging System. Band intensity was expressed as a ratio of phospho/total for each protein band of interest, and for each treated sample these ratios were then expressed as a percentage of the control (untreated) samples run on the same gel. Each control value was taken as the average of 2–3 identically treated samples on each gel.

### 2.4 ROS measurement

PASMC were seeded into 96-well plates, grown to 80–90% confluence and serum-starved for 24hrs. Cell were then incubated with the luminol-derived superoxide probe 8-amino-5-chloro-7-phenylpyrido[3,4-*d*]pyridazine-1,4-(2 H,3 H)-dione (L-012, 50 µM, Wako Pure Chemical Industries) [27], in PBS at 37 °C. We also used an alternative chemi-luminescence ROS probe (ROS-Glo™, Promega) [28] to measure H_2_O_2_ production in IPA. Changes in luminescence induced by acute drug treatments were detected using a Promega GloMax Multi+ plate reader. Background measurements using ROS probe plus drug, but in the absence of cells/tissue, were subtracted from all equivalent test readings in the presence of cells/tissue. Each final value for each treatment for each batch of cells was the average of measurements from at least 8 identically treated wells in each plate.

### 2.5 c-Src oxidation assay

Reversible oxidation of c-Src cysteine residues in response to acute treatment with U46619 or exogenous ROS was determined using the semi-quantitative PEG-switch method [29]. Briefly, this involves incubating IPA lysates with maleimide (100 mM, 25 min, 50 °C) to alkylate reduced cysteine residues. Then, following reduction of reversibly oxidised cysteines with dithiothreitol (DTT, 200 mM, 30 min, RT°C) and desalting to remove maleimide and DTT (Zeba Spin desalting columns, Thermo Scientific), lysates are incubated with polyethyleneglycol 5000-tagged maleimide (PEG-maleimide, 10 mM, 2 h, RT°C), to alkylate remaining reduced cysteine residues. When subjecting the lysate to SDS-PAGE and probing for c-Src by Western blot, reversible oxidation is indicated by separation of c-Src into two or more bands due to oxidised cysteine pegylation increasing the protein molecular weight by multiples of 5KDa.

### 2.6 cDNA cloning, siRNA design and cell transfection

Rat RhoA cDNA was cloned into PCR2.1-TOPO^®^ TA vector (Invitrogen Life Technologies, UK) by PCR using reverse transcribed RNA that was obtained from PASMCs, whereas rat ARHGEF1 cDNA was obtained from Source Bioscience UK Ltd. These were then used as templates for cloning into pcDNA™6.2/C-EmGFP/TOPO® vector (Invitrogen Life Technologies, UK). Oligonucleotide primers were designed in accordance with the manual and using sequences form the GenBank^®^ database (RhoA accession no. BC061732; ARHGEF1 accession no. BC091218) as follows: Rat RhoA (forward): GTTATGGCTGCCATCAGGAAGAAACTGG-3′, Rat RhoA (reverse): 5′-CAAGATGAGGCACCCCGACT-3′, Rat ARHGEF1 (forward): 5′-GAGATGGGAGAAGTCGCCGGAGGGGC-3′, Rat ARHGEF1 (reverse): 5′-TGAAAGGCCTGTCTGAGCAGAGCGC-3′. The stop codon is intentionally missed in the reverse primer to allow for fusion of the EmGFP sequence that is present in the vector. The TOPO^®^ TA technology will result in cloning of PCR products in both forward and reverse orientations, therefore clones were initially checked for the correct orientation using restriction endonuclease digestion and gel electrophoresis. These were then fully checked by sequencing (Source BioScience UK Limited) for selection. The rat ARHGEF1 siRNA was designed as described previously [11], [30]. The 19 nucleotide target sequence (position 389–407, GenBank accession no. BC091218) was synthesized into 64–65 mer oligonucleotides with *Bam*HI/*Hin*dIII overhangs (Sigma) and cloned into the expression vector pSilencer 3.0-H1 (Ambion Inc.). All clones were purified (EndoFree Plasmid Maxi Kit, Qiagen Ltd) and sequenced (Source BioScience UK Limited). A control siRNA was designed based on a scrambled version of the ARHGEF1 target sequence.

Transfection was carried out in detached confluent PASMCs by electroporation using Amaxa™ Basic Nucleofector™ Kit for Primary Smooth Muscle Cells (LONZA) and a Nucleofector™ 2b Device (Lonza). Transfected cells were re-suspended in DMEM, seeded onto 19 mm glass coverslips (VWR international), grown to ~50% confluence and serum-starved for 24 h. In each batch of cells transfected with ARHGEF1 siRNA, efficient knock-down of ARHGEF1 protein was confirmed by Western Blot (78 ± 3% reduction vs. GAPDH, n=3 transfections).

### 2.7 Cell imaging and quantification

Coverslips were mounted onto a Zeiss Axiovert 200 Microscope and cells visualised using BD™ CARV II Confocal Imager under X40 magnification, bathed in PSS/5% CO_2_ at 37 °C. When illuminated with UV/blue light (~390 nm). Successfully transfected cells were identified by bright green fluorescence. To minimise EmGFP bleaching, illumination was limited to regular but brief (65 ms) shutter openings: typically once every 2 s during agonist stimulation, but less frequent during rest periods. During each illumination, images were captured by MetaFluor v.7 software (Molecular Devices). Regional and temporal changes in fluorescence in response to drug treatments were quantified using ImageJ software (rsb.info.nih.gov).

### 2.8 Pharmacological agents, antioxidants and antibodies

The Src-family kinase inhibitor 4-amino-5-(4-chlorophenyl)-7-(t-butyl)pyrazolo[3,4-*d*]pyrimidine (PP2), the antioxidants ebselen and hydroxy-tempo (Tempol), the intracellular superoxide generator 6-anilo-5,8-quinoledione (LY83583) and the thromboxane A2 mimetic U46619 were all obtained from Merck Millipore. Superoxide dismutase (SOD), catalase, maleimide, polyethyleneglycol maleimide 5000, dithiothreitol, the nitric oxide synthase inhibitor L-nitro-arginine methyl-ester (L-NAME) and the inhibitor of RGS-domain containing RhoGEFs (Y16) were obtained from Sigma. Anti-Src, anti-phospho-Src family kinases (Tyr416), anti-MLC_20_, anti-phospho-MLC_20_ (Ser19), anti-MYPT1, anti-ARHGEF1, anti-phospho-tyrosine (PY100) and anti-GAPDH were all obtained from Cell Signalling (UK). Anti-phospho-MYPT1 (Thr850) was from Upstate (UK). Anti-mouse IgG and anti-rabbit IgG were from Sigma.

### 2.9 Statistical analysis

Statistical analysis was performed using SigmaPlot (Systat Software Inc.). Two group comparisons were by paired or un-paired *t*-test where appropriate. 1 factor or 2 factor multiple group comparisons were by 1-way or 2-way ANOVA respectively, with appropriate post-tests. Unless otherwise stated, n is the number of animals or cell lines derived from individual animals. All values are expressed as mean ± SEM.

## 3 Results

### 3.1 Endogenous ROS and SrcFK contribute to U46619-induced contraction

**Fig. 1:**
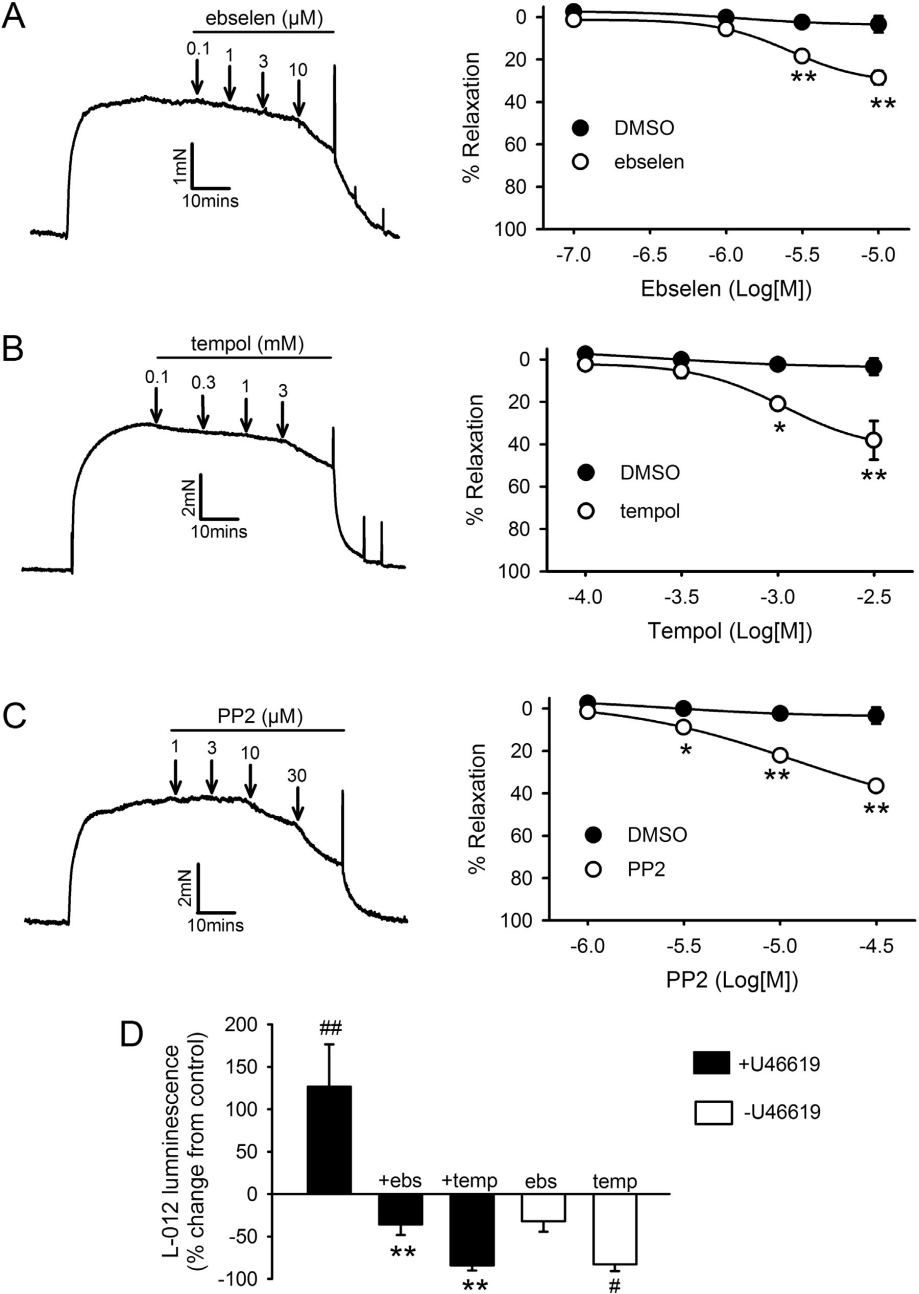
Effects of U46619, antioxidants and SrcFK inhibition on contraction and ROS production. **A-C**: Concentration-dependent relaxation responses to ebselen (A, n=8), Tempol (B, n=7) and PP2 (C, n=6) in IPA pre-constricted with 100 nM U46619. Left panels: representative traces with arrows indicating where each dose was added. Right panels: mean effects of inhibitors plotted against DMSO vehicle control (n=5). *P<0.05, **P<0.01 vs. DMSO control (2-way ANOVA). **D:** Effect of ebselen (10 µM) or Tempol (3 mM) on U46619-induced ROS production (15 min, 100 nM, solid bars) or on basal ROS production (open bars) in PASMC, measured by L-012 chemi-luminescence. #P<0.01 vs. control; ##P<0.001 vs. control; **P<0.001 vs. U46619 alone (2-way ANOVA, n=10).

To determine the contribution of endogenous ROS to agonist-induced contraction, we examined the effects of the two structurally dissimilar non-selective antioxidants ebselen and Tempol [31], [32] on contractile responses induced by the thromboxane mimetic U46619 in IPA. 100 nM U46619 produced submaximal contractions equivalent to 50–75% of KPSS. Both inhibitors induced concentration-dependent relaxation (30–40% relaxation, Fig. 1A-B). As shown previously with another TP receptor agonist PGF_2α_ [24], U46619-induced contraction was also sensitive to inhibition by the SrcFK antagonist PP2, which induced concentration-dependent relaxation (~40% relaxation, Fig. 1C). In order to eliminate the role of nitric oxide in the effects of antioxidants and PP2 on U46619-induced contraction, these experiments were repeated in the presence of 300 µM L-NAME. Under these conditions, relaxation responses to ebselen (n=6), Tempol (n=6) and PP2 (n=7) were essentially the same as without L-NAME (Supplementary Fig. S1). The effect of Tempol and ebselen on KPSS-induced contraction was indistinguishable from that of vehicle (Tempol, 7.7 ± 2.8% block at 3 mM, n=8; ebselen, 10 ± 2% block at 10 µM, n=8, DMSO, 7.2 ± 2.1% block, n=6).

We then examined the effect of U46619 on endogenous ROS production in PASMC using the Luminol-derived ROS-sensitive chemi-luminescent probe L-012 [27]. U46619 caused a marked increase in superoxide-selective L-012 chemi-luminescence and this increase was abolished by prior incubation with ebselen or Tempol (Fig. 1D). Basal chemi-luminescence was all but abolished by Tempol, while ebselen was without significant effect (Fig. 1D). U46619 also enhanced H_2_O_2_-selective chemi-luminescence in IPA as measured using the Promega ROS-Glo™ H_2_O_2_ assay (196 ± 19.4% increase from control, n=3).

### 3.2 Exogenous ROS cause contraction in a SrcFK-dependent manner

**Fig. 2:**
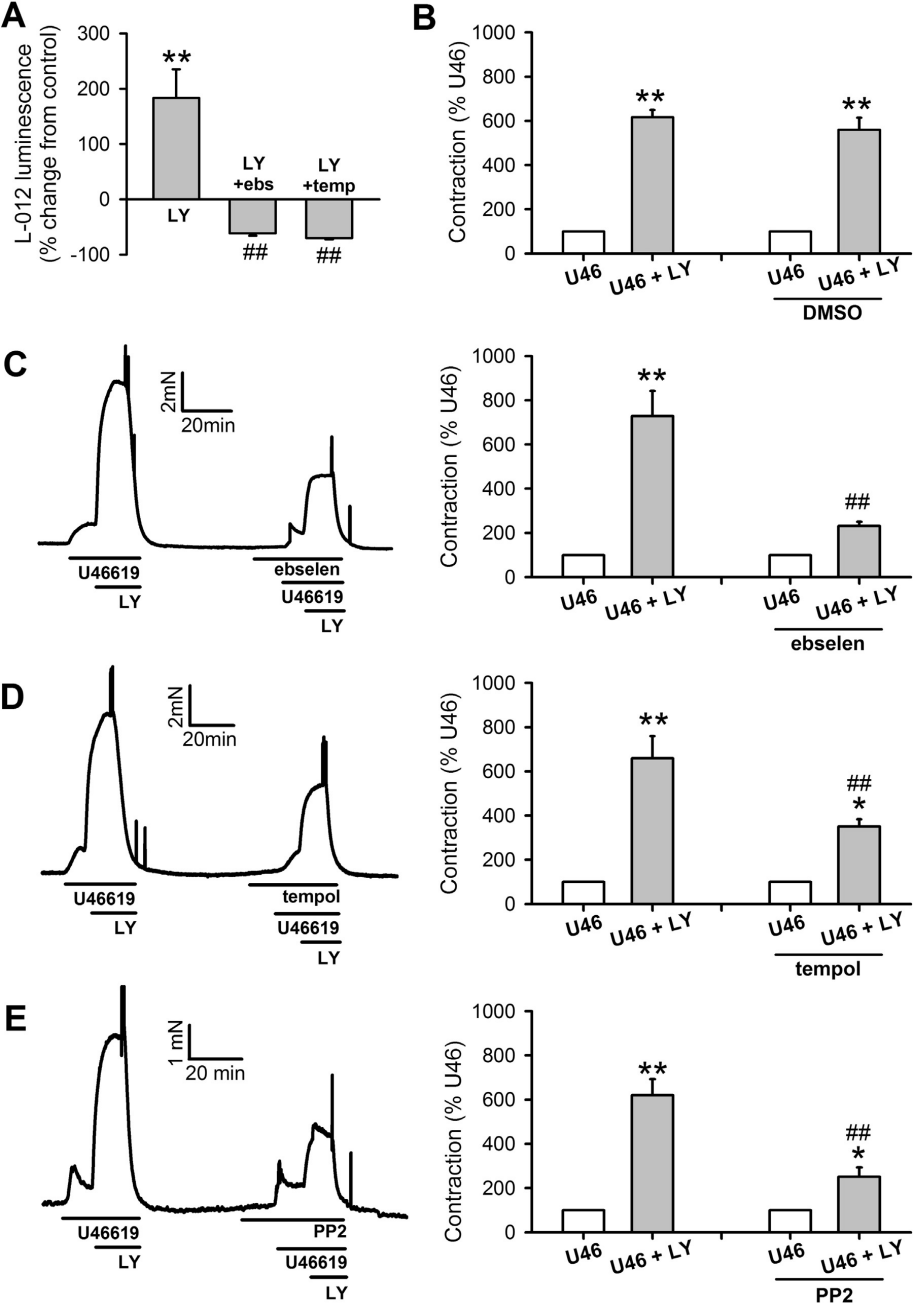
Effects of exogenous superoxide on U46619-induced contraction in IPA. **A:** LY83583 (LY, 1 µM, 15 min, n=12) generates superoxide in PASMC, as measured by L-012 chemi-luminescence), and this is inhibited by ebselen (ebs, 10 µM, n=10) or Tempol (temp, 3 mM, n=10). **P<0.001 vs. control, ##P<0.001 vs. LY alone. **B-E**: Effects of LY83583 and inhibitors on U46619-induced contraction in IPA. U46619 concentration was adjusted in each case to produce contractions equivalent to ~10% of KPSS (U46). 1 µM LY83583 was then added for 10 min (U46+LY). This response was then repeated in the presence of either DMSO vehicle (**B**, n=6), ebselen (**C**, 10 µM, n=7), Tempol (**D**, 3 mM, n=11) or PP2 (**E**, 30 µM, n=8). Left panels: representative traces. Right panels: mean ± SEM measurements of peak LY83583-induced contraction, expressed as a percentage of the U46619 pre-constriction. *P<0.05 vs. U46; **P<0.01 vs. U46, ##P<0.001 vs. control U46+LY (2-way ANOVA).

As expected, the membrane permeable quinolinequinone LY83583, a known generator of intracellular superoxide [15], [33], [34], increased L-012 chemi-luminescence, and at 1 µM this increase was similar to that observed with 100 nM U46619 and was similarly abolished by ebselen and Tempol (Fig. 2A). While higher doses of LY83583 can directly constrict IPA [15], 1 µM LY83583 does not itself cause contraction, but causes a several-fold enhancement of the response to 2 nM U46619, which is initially ~10% of KPSS in amplitude. When this response was repeated in the same arteries, but in the presence of ebselen, Tempol or PP2, with U46619 concentration at 8–10 nM to match pre-constriction amplitude, the enhancing effect of LY83583 was significantly reduced, while DMSO was without effect (Figs. 2B-2E). To eliminate the possibility that the enhancing effect of LY83583 on U46619-induced contraction was as a result of nitric oxide scavenging by superoxide, responses were repeated in the presence of L-NAME (300 µM, n=6). Under these conditions, enhancement of the U46619-induced contraction was essentially the same as without L-NAME (Supplementary Fig. S1). 30 µM H_2_O_2_ also caused contraction in IPA and this contraction was partially sensitive to inhibition by PP2 (Supplementary Fig. S2A).

To determine the relative contribution of superoxide and H_2_O_2_ to U46619-induced contraction, contraction responses to U46619 were repeated after prior incubation with either catalase alone, to remove H_2_O_2_, or catalase combined with superoxide dismutase (SOD), to remove both superoxide and H_2_O_2_. As shown in Supplementary Fig. S3 A-B, catalase alone partially but significantly inhibited contraction, while the combined effect of catalase and SOD caused an even greater degree of inhibition that was significantly greater than catalase alone.

### 3.3 U46619 and exogenous ROS stimulate c-Src cysteine oxidation, ROS-dependent SrcFK activity and tyrosine phosphorylation in IPA

**Fig. 3:**
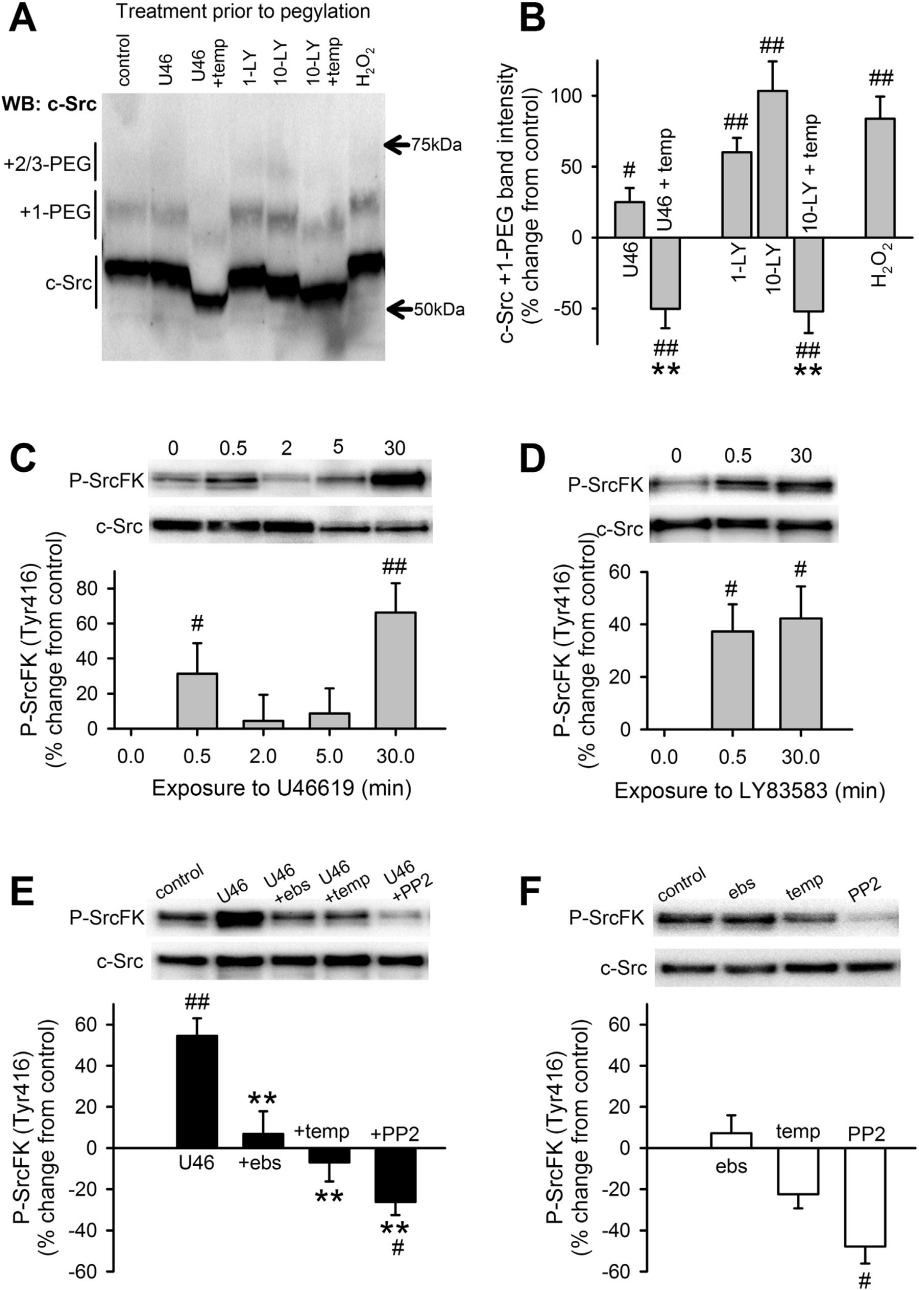
Effects of U46619, antioxidants and exogenous ROS on c-Src cysteine oxidation, and SrcFK auto-phosphorylation in IPA. **A, B:** c-Src cysteine oxidation in IPA is significantly enhanced by U46619 (U46, 100 nM, 30 min, n=9), LY83583 (LY, 1 or 10 µM, 30 min, n=7–8) and H_2_O_2_ (30 µM, 30 min, n=5). This enhancement and part of the underlying basal oxidation, is prevented by Tempol (temp, 3 mM, n=6–8). #P<0.05, ##P<0.01 vs. control, **P<0.01 vs. U46 only or vs. 10-LY only (1-way ANOVA). **C:** U46619 (100 nM) time-dependently enhances SrcK auto-phosphorylation (Tyr416) in IPA (n=9, #P<0.05 vs. control, ##P<0.01 vs. control, 1-way ANOVA). **D:** In the absence of U46, SrcFK auto-phosphorylation is significantly enhanced by LY83583 (1 µM) at 0.5 min (n=16) and 30 min (n=7), #P<0.05 vs. control (1-way ANOVA). **E:** At 30 min, U46619-induced SrcFK auto-phosphorylation is inhibited by ebselen (ebs, 10 µM, n=14), Tempol (n=11) or PP2 (30 µM, n=10) #P<0.05 vs. control. **F:** In the absence of U46, SrcFK auto-phosphorylation is unaffected by ebselen (n=10) or Tempol (n=10) but significantly inhibited by PP2 (n=8), ##P<0.001 vs. control, **P<0.001 vs. U46 alone (n=19, 2-way ANOVA).

Having established that U46619 enhances PASMC ROS production and that U46619-induced contraction in IPA is dependent on both ROS and SrcFK, we next investigated whether there was a causative link between ROS and SrcFK activity. After subjecting IPA lysates to the PEG-switch protocol [29] for the visualisation of reversible cysteine oxidation, probing for c-Src revealed additional bands on the Western blot. The most prominent of these was approximately 5 kDa larger than the main band (designated c-Src +1-PEG) indicative of oxidation of a single cysteine residue (Fig. 3A). Although this second band was present in all samples, its intensity was treatment-specific, being slightly but significantly enhanced by 100 nM U46619, 1 and 10 µM LY83583 and 30 µM H_2_O_2_, and suppressed by Tempol (Fig. 3A, B). Tempol reduced band intensity significantly below control levels, suggesting a degree of basal c-Src oxidation. A third band, 10–15 kDa above the main c-Src band was also present in some samples, indicative of multiple cysteine oxidation (designated c-Src +2/3-PEG), but was too weak for quantification. Intensity of the main band (non-oxidised c-Src) was minimally affected by all treatments applied (Supplementary Fig. S4A). Control experiments confirm that additional bands indicative of cysteine pegylation are absent when the PEG-maleimide incubation step is omitted or when the de-salting step alone is performed (Supplementary Fig. S4B).

U46619 induced biphasic time-dependent increases in SrcFK auto-phosphorylation (on Tyr416) with peaks at 0.5 min and 30 min (Fig. 3C). LY83583 and exogenous H_2_O_2_ also enhanced SrcFK auto-phosphorylation in the absence of U46619, suggesting that SrcFK activity is ROS sensitive (Fig. 3D, Supplementary Fig. S2B). To corroborate this, the U46619-induced increase at 30 min was inhibited by prior incubation with ebselen, Tempol or PP2 (Fig. 3E), while basal SrcFK phosphorylation was unaffected by ebselen, marginally reduced by Tempol, but only significantly inhibited by PP2 (Fig. 3F). To illustrate the relative contribution of superoxide and H_2_O_2_ to U46619-induced phosphorylation responses, U46619-induced SrcFK auto-phosphorylation at 30 min was also partially inhibited by catalase and further inhibited by the combined action of SOD and catalase, while basal phosphorylation was unaffected by either condition (Supplementary Fig. S3, C-D).

When blots were probed with anti-phospho-tyrosine to visualise tyrosine phosphorylation of multiple proteins, it was evident that LY83583 enhanced this phosphorylation at multiple bands, including those with approximate MW of 80, 90 and 115 kDa. These three bands and another band at approximately 140 kDa were sensitive to PP2, indicative of ROS-induced, SrcFK-dependent phosphorylation (Supplementary Fig. S5). Taken together, these results suggest a link between ROS production, SrcFK activation and tyrosine phosphorylation.

### 3.4 U46619 stimulates MYPT-1 and MLC_20_ phosphorylation in IPA in a ROS-dependent and SrcFK-dependent manner

**Fig. 4:**
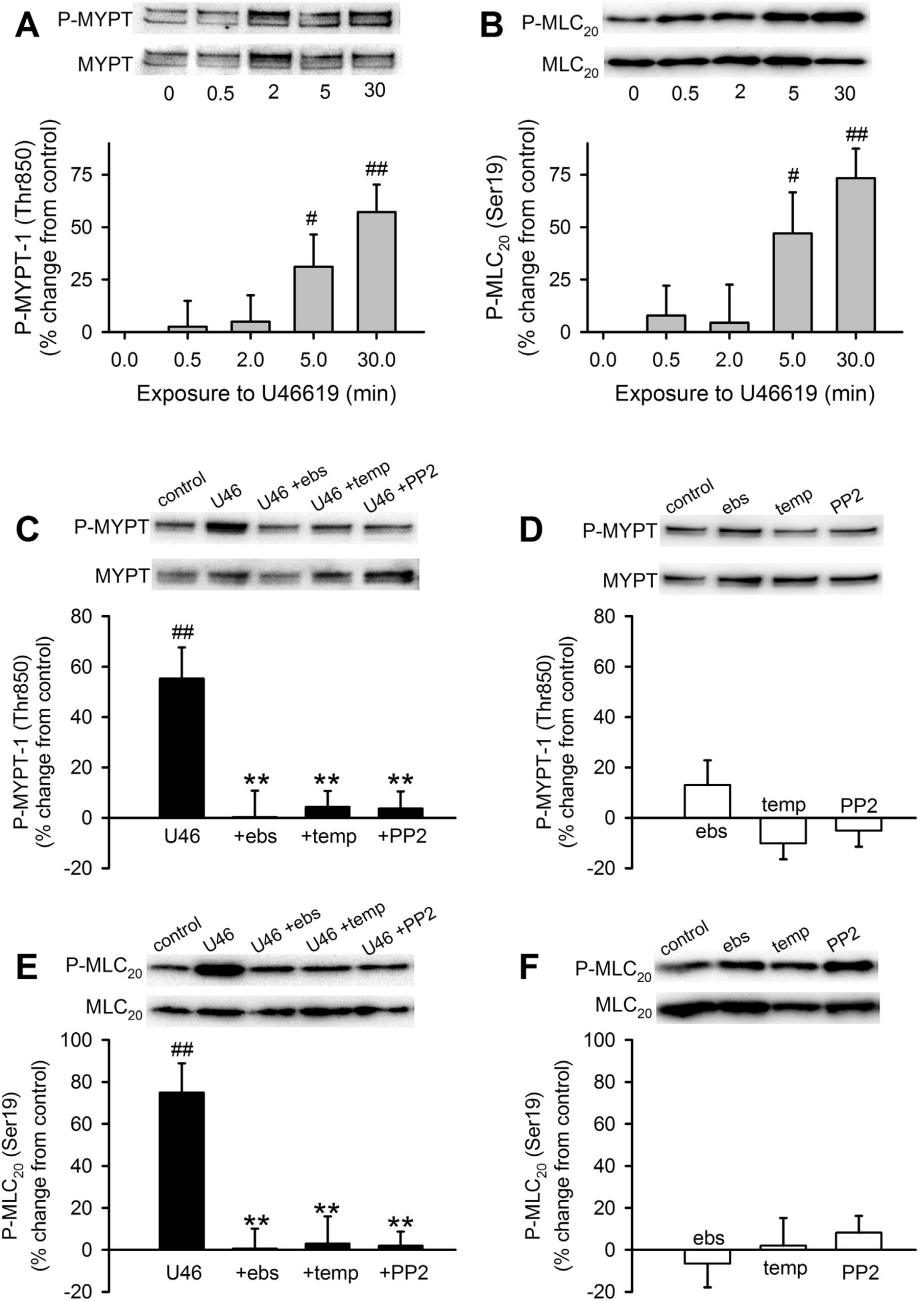
Contribution of ROS and SrcFK to U46619-induced Rho-kinase activity and MLC_20_ phosphorylation in IPA. U46619 (U46, 100 nM) time-dependently enhances phosphorylation of MYPT-1 on Thr850 (**A**), and of MLC_20_ on Ser19 (**B**), in IPA (n=8–10, #P<0.05 vs. control, ##P<0.001 vs. control, 1-way ANOVA). **C:** At 30 min, U46619-induced MYPT-1 phosphorylation is inhibited by ebselen (ebs, 10 µM, n=8), Tempol (temp, 3 mM, n=12) and PP2 (30 µM, n=18) ##P<0.001 vs. control (n=27), **P<0.001 vs. U46 alone, while in the absence of U46 (**D**), MYPT-1 phosphorylation was unaffected by ebselen (n=11), Tempol (n=12) or PP2 (n=18) (2-way ANOVA). **E:** Similarly, at 30 min, U46619-induced MLC_20_ phosphorylation is also inhibited by ebs (n=14), temp (n=12) and PP2 (n=17) ##P<0.001 vs. control (n=27), **P<0.001 vs. U46 alone, while in the absence of U46 (**F**), MLC_20_ phosphorylation is unaffected by ebselen (n=13), Tempol (n=12) or PP2 (n=18) (2-way ANOVA).

We reported previously that SrcFK contribute to pulmonary artery contraction induced by a GPCR agonist (PGF_2α_) or hypoxia in part by activating Rho-kinase [11], [24] and that superoxide generated by LY83583 also activates Rho-kinase [15]. To determine whether U46619-induced activation of Rho-kinase and myosin light-chain kinase (MLCK) was dependent on both *endogenous* ROS and SrcFK, we examined phosphorylation of the Rho-kinase target myosin phosphatase targeting subunit-1 (MYPT-1, Thr850) and of myosin light-chain-20 (MLC_20_, Ser19), the target of MLCK. U46619 triggered time-dependent increases in phosphorylation of both MYPT-1 (Fig. 4A) and MLC_20_ (Fig. 4B). At 30 min, these increases were completely inhibited by prior incubation with either ebselen, Tempol or PP2 (MYPT-1: Fig. 4C, MLC_20_: Fig. 4E), while in the absence of U46619, basal phosphorylation of either protein was not significantly altered by any of the three inhibitors (Fig. 4D, F). To illustrate the relative contribution of superoxide and H_2_O_2_ to U46619-induced phosphorylation, both MYPT-1 and MLC_20_ phosphorylations induced by U46619 at 30 min were partially inhibited by catalase and further inhibited by the combined action of SOD and catalase, while neither condition affected basal phosphorylation of either protein (Supplementary Fig. S3, E-H).

### 3.5 Hypoxia-induced SrcFK, MYPT-1 and MLC_20_ phosphorylation is dependent on mitochondrial ETC complex I & III

**Fig. 5:**
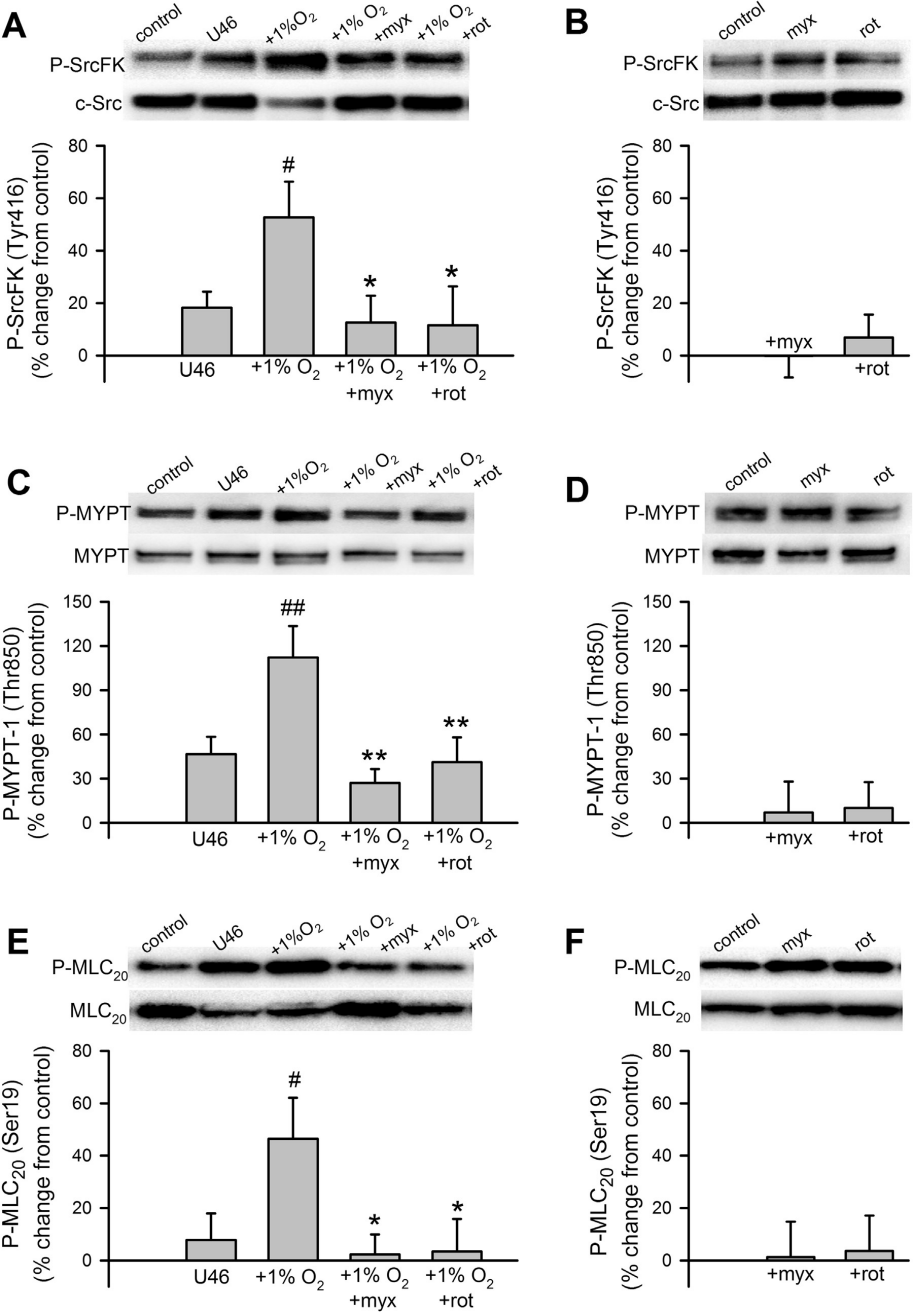
Effects of hypoxia and mitochondrial ETC inhibitors on SrcFK, MYPT-1 and MLC_20_ phosphorylation in IPA. **A:** Hypoxia (1% O_2_, 1 min) enhances SrcFK auto-phosphorylation following priming with U46619 (U46, 1 nM, 15 min), and this enhancement is prevented by myxothiazol (myx, 100 nM) or rotenone (rot, 100 nM). #P<0.05 vs. U46 alone, *P<0.05 vs. U46/1% O_2_ (2-way ANOVA, n=9–10). **B:** Myxothiazol alone or rotenone alone has no significant effect on SrcFK auto-phosphorylation (1-way ANOVA, n=8–9). **C**: Hypoxia (1% O_2_, 5 min) enhances MYPT-1 phosphorylation following priming with U46619 (U46, 1 nM, 15 min), and this enhancement is prevented by myxothiazol (myx, 100 nM) or rotenone (rot, 100 nM). ##P<0.01 vs. U46 alone, **P<0.01 vs. U46/1% O_2_ (2-way ANOVA, n=9–10). **D:** Myxothiazol or rotenone alone (in the absence of contractile stimuli) have no significant effect on MYPT-1 phosphorylation (1-way ANOVA, n=9–11). **E**: Hypoxia (1% O_2_, 5 min) enhances MLC_20_ phosphorylation following priming with U46619 (U46, 1 nM, 15 min), and this enhancement is prevented by myxothiazol (myx, 100 nM) or rotenone (rot, 100 nM) #P<0.05 vs. U46 alone, *P<0.05 vs. U46/1% O_2_ (2-way ANOVA, n=9–10). **F:** Myxothiazol or rotenone alone (in the absence of contractile stimuli) have no significant effect on MLC_20_ phosphorylation (1-way ANOVA, n=9–10).

To determine whether hypoxia-induced SrcFK and Rho-kinase activity in IPA is dependent on mitochondrial ETC ROS production, we evaluated the effects of rotenone and myxothiazol, inhibitors of the source of mitochondrial ROS (complex I and III, respectively) on peak hypoxia-induced SrcFK (1 min), MYPT-1 (5 min) and MLC_20_ (5 min) phosphorylation responses in IPA. Since a small degree of pre-constriction is required for a full hypoxic contractile response in IPA [35], phosphorylation responses were similarly evaluated after priming with 1 nM U46619 for 15 min. Hypoxia enhanced phosphorylation of all three proteins, and this enhancement was abolished by either rotenone or myxothiazol (Fig. 5A, C, E). Conversely, the underlying U46619-induced phosphorylation was largely unaffected by rotenone or myxothiazol and basal phosphorylation totally insensitive to either inhibitor (Fig. 5B, D, F). U46619-induced contraction (100 nM, 15 min) was not significantly inhibited by pre-incubation with either rotenone (control 46 ± 14% of KPSS vs. rot 44 ± 15% of KPSS, n=6, paired *t*-test) or myxothiazol (control 42 ± 7% of KPSS vs. myx 30 ± 4% of KPSS, n=8, paired *t*-test).

### 3.6 U46619 induces ROS-dependent and SrcFK-dependent translocation of RhoA and ARHGEF-1

**Fig. 6:**
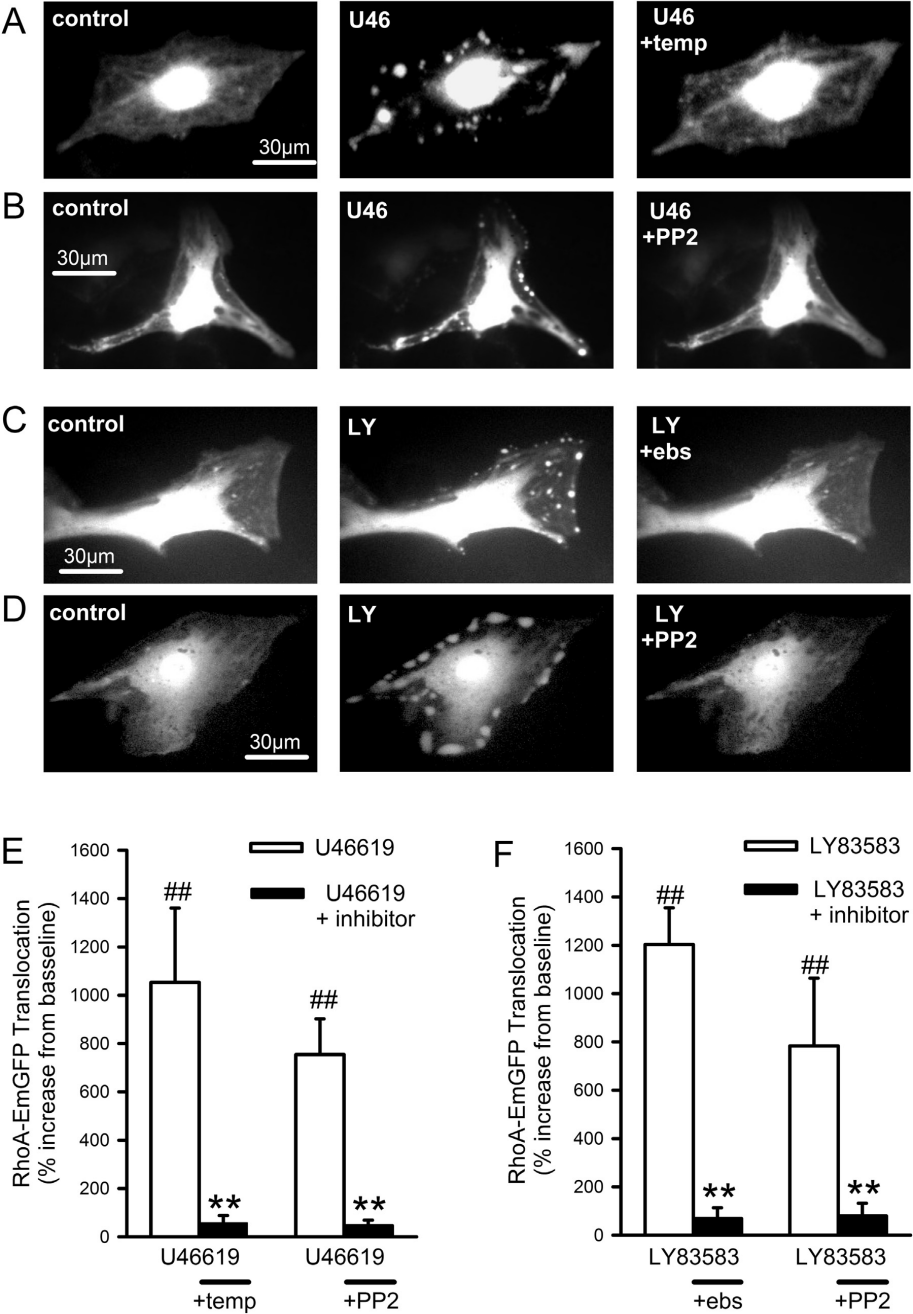
Effects of U46619, LY83583, antioxidants and SrcFK inhibition on RhoA-EmGFP translocation in PASMC. **A-D:** Fluorescent imaging of representative RhoA-EmGFP transfected PASMC at rest (left panels), in the presence of stimulus (middle panels **A-B**: U46619, U46, 100 nM or **C-D**: LY83583, LY, 1 µM) or after washout followed by repeated exposure to stimulus in the presence of either Tempol, PP2 or ebselen (right panels). **E-F:** Quantification of spot/patch fluorescence intensity expressed as % change above control levels during stimulus ± inhibition (**E**: U46619, **F**: LY83583). ##P<0.001 vs. baseline, **P<0.001 vs. U46619, 2-way ANOVA, n=8–11 cells from a total of seven different cell lines. Each measurement is combined from at least 3 spots/patches from each cell. See supplementary Fig. S6 for control responses.

Rho-kinase activation requires prior activation of the monomeric G-protein RhoA, and RhoA activation is often associated with its translocation from the cytosol to the plasma membrane [36], [37]. We therefore visualised and quantified translocation of EmGFP-tagged RhoA in live PASMC, as a corollary of RhoA activation, in response to U46619 and LY83583. Under resting conditions, RhoA-EmGFP fluorescence was localised primarily in the nucleus/perinuclear region, with additional lower level fluorescence diffusely distributed over the rest of the cell. In the majority of transfected cells examined (>60%), stimulation with either U46619 or LY83583 triggered the rapid appearance of highly localised bright spots or patches on the cell periphery or at prominent cellular attachment points. RhoA translocation was fully reversible upon washout. Following an initial response and washout (5 min), cells were then incubated for 5 min with ebselen, Tempol, PP2 or DMSO before being re-exposed to either U46619 or LY83583. The second translocation responses were greatly diminished or abolished by all three inhibitors, but unaffected by DMSO (Fig. 6, Fig. S6). It was also observed that, upon application of U46619 or LY83583, the reversible appearance of bright peripheral spots or patches was associated with concomitant reversible darkening of surrounding cytosolic fluorescence (Fig. 6, Fig. S9). These changes were confirmed by quantification (Fig. S9), further supporting the assumption that RhoA-EmGFP was indeed translocating in response to stimulus.

**Fig. 7:**
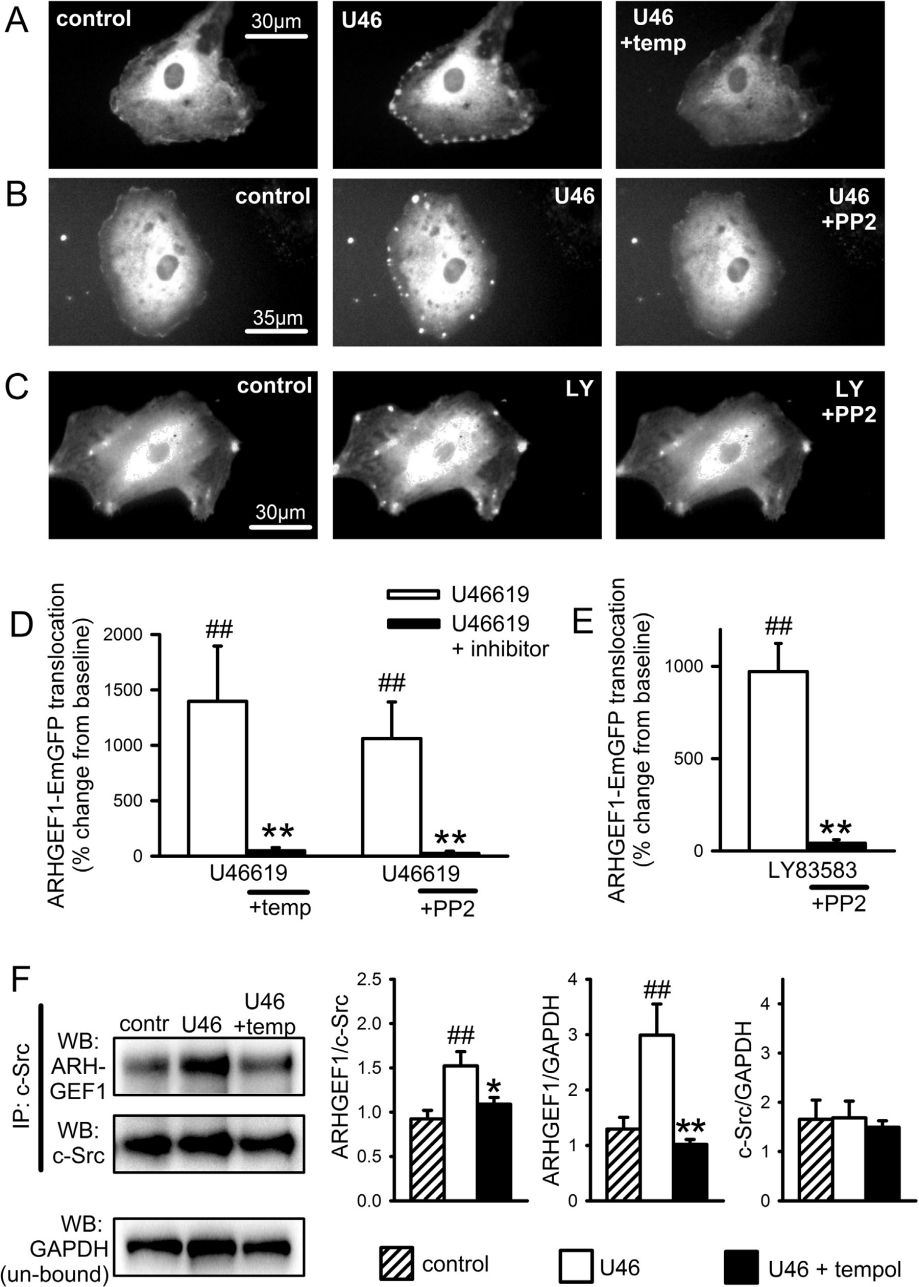
ARGHEF1-EmGFP translocates in response to U46619 and LY83583 in PASMC and co-localises with c-Src in IPA. **A-C:** Fluorescent imaging of representative ARHGEF1-EmGFP transfected PASMC at rest (left panels), in the presence of stimulus (middle panels **A-B**: U46619, U46, 100 nM or **C**: LY83583, LY, 1 µM) or after washout followed by repeated exposure to stimulus in the presence of either Tempol or PP2 (right panels). **D-E:** Quantification of spot/patch fluorescence intensity expressed as % change above control levels during stimulus ± inhibition (**D**: U46619, **E**: LY83583). ##P<0.001 vs. baseline, **P<0.001 vs. U46619, 2-way ANOVA, n=8–9 cells from a total of seven different cell lines. Each measurement is combined from at least 3 spots/patches from each cell. See supplementary Fig. S8 for control responses. **F**: Co-localisation of ARHGEF1 with c-Src in IPA. Representative blots show the effect of U46619 (100 nM, 30 min) on ARHGEF1 content (WB: GEF1) and c-Src content (WB: c-Src) after c-Src immuno-precipitation (IP: c-Src), with GAPDH content in the un-bound fraction as a loading control (WB: GAPDH). Bar charts show that U46619 treatment increases ARHGEF1 co-immuno-precipitation with c-Src and that this is prevented by pre-incubation with Tempol (3 mM). ARHGEF1 content expressed relative to both c-Src IP content and GAPDH loading control. c-Src IP content is unaffected by treatment. ##P<0.0.1 vs. control, *P<0.05, **P<0.01 vs. U46, 1-way ANOVA, n=6.

Hypothesising that ROS and SrcFK may be mediating RhoA translocation and Rho-kinase activity via interaction with one or more RhoGEFs, we also examined the involvement of ARHGEF1, a RhoA-specific guanine nucleotide exchange factor. Expression of native ARHGEF1 in IPA was confirmed by PCR (Fig. S7). In parallel with RhoA-EmGFP translocation, we also examined the translocation responses of EmGFP-tagged ARHGEF1 in live PASMC, in response to U46619 and LY83583. ARHGEF1-EmGFP responded to both stimuli with a pattern of translocation similar to that of RhoA-EmGFP, being similarly stimulated by U46619 or LY83583 and similarly inhibited by Tempol, ebselen or PP2 (Fig. 7A-E, Figs. S8 & S9), although it was noticeably absent from nuclei and less persistent than for RhoA, fading after several seconds. To further clarify the relationship between SrcFK and ARHGEF1, we also examined their physical association in IPA and its ROS-dependence by co-immunoprecipitation. As shown in Fig. 7F, ARHGEF1 was co-immunoprecipitated with c-Src and this co-immunoprecipitation was enhanced by U46619 and prevented by the antioxidant Tempol.

### 3.7 RhoA translocation and contraction induced by U46619 and LY83583 require RhoGEF activity

**Fig. 8:**
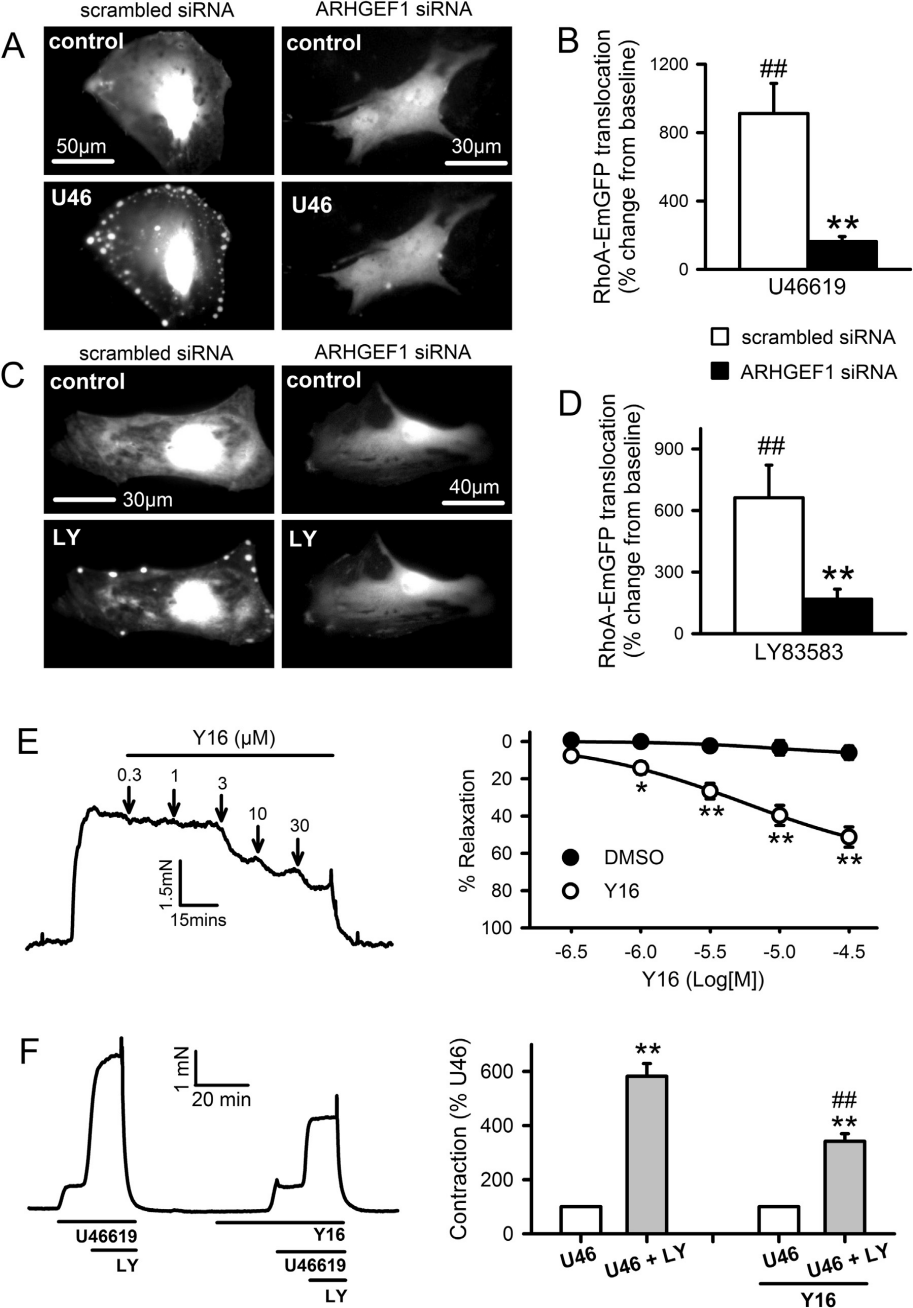
Effects of RhoGEF inhibition on U46619 and ROS-induced RhoA translocation and contraction. **A-D**: Fluorescence imaging of RhoA-EmGFP translocation in PASMC, co-transfected with ARHGEF1 siRNA or scrambled siRNA, stimulated with U46619 (**A**: U46, 100 nM) or LY83583 (**C**: LY, 1 µM). Quantification of spot/patch fluorescence intensity expressed as % change above control levels during stimulus ± inhibition (**B**: U46619, **D**: LY83583). ##P<0.001 vs. baseline, **P<0.001 vs. treatment, 2-way ANOVA, n=12–16 cells from a total of three different cell lines. Each measurement is combined from at least 3 spots/patches from each cell. **E**: Concentration-dependent relaxation responses Y16 (10 µM) in IPA pre-constricted with 100 nM U46619. Left panels: representative traces with arrows indicating where each dose was added. Right panels: mean effects of inhibitor plotted against DMSO vehicle control (n=12). *P<0.05, **P<0.01 vs. DMSO control (2-way ANOVA). **F**: Effects of LY83583 and Y16 on U46619-induced contraction in IPA. U46619 concentration was adjusted in each case to produce contractions equivalent to ~10% of KPSS. 1 µM LY83583 was then added for 10 min (U46+LY). This response was then repeated in the presence of Y16 (10 µM, n=13). Left panels: representative traces. Right panels: mean ± SEM measurements of peak LY83583-induced contraction, expressed as a percentage of the U46619 pre-constriction. **P<0.01 vs. U46, ##P<0.001 vs. control U46+LY (2-way ANOVA).

If ARHGEF1 translocates and associates with c-Src in response to contractile stimuli, it is possible that activation of RhoA and contraction are also dependent on ARHGEF1. We first tested this hypothesis by examining translocation of RhoA-EmGFP in PASMC co-transfected with an siRNA against ARHGEF1. Secondly, we determined the effects of Y16 on U46619 and LY83583-induced contraction. Y16 is an inhibitor of the RGS domain-containing sub-family of RhoA-specific RhoGEFs, which includes ARHGEF1 [38]. In PASMC co-transfected with a control (scrambled) siRNA, both U46619 and LY83583 triggered translocation of RhoA-EmGFP in a manner similar to that described in Fig. 6. In contrast, this translocation was nearly absent in PASMC co-transfected with ARHGEF1 siRNA (Fig. 8A-D). In IPA pre-constricted with 100 nM U46619, Y16 caused concentration-dependent relaxation (Fig. 8E) and in IPA contracted by 1 µM LY83583 following pre-constricted with low-dose U46619 (<20 nM), the LY83583-induced enhancement was significantly inhibited by 10 µM Y16 (Fig. 8F). Thus, ROS-dependent contraction of IPA requires the activation of a RhoGEF, probably ARHGEF1, upstream of RhoA/Rho-kinase.

## 4 Discussion

It is generally accepted that ROS are important signalling molecules in vascular function. However, their involvement in normal vascular smooth muscle contractile responses in general, and Rho-kinase activation in particular, remains to be fully characterised. We hypothesised that SrcFK act as proximal ROS-activated molecular switches in rat IPA, responding to endogenous and exogenous ROS signals in order to activate Rho-kinase and contribute to contraction. We also sought to link SrcFK to Rho-kinase via RhoGEF and RhoA activation in this model. Here we showed that U46619, an agonist for the G-protein coupled T-type prostanoid receptor, enhances ROS production in PASMC, and as expected, antioxidants fully inhibited U46619-induced ROS production. Although we haven’t determined the source of ROS in this study, it is most likely derived from NADPH oxidase, as has been previously described for U46619 and other agonists in this tissue [1], [39], as well as for multiple other G-protein coupled receptor agonists in various other vascular preparations [40], [41], [42].

### 4.1 Sequential activation of SrcFK and Rho-kinase by endogenous and exogenous ROS

SrcFK auto-phosphorylation is a recognised indicator of SrcFK activity [43], while the phosphorylation of MYPT-1 on Thr850 by Rho-kinase results in disassembly of myosin light-chain phosphatase [44]. We showed that the two structurally unrelated non-selective antioxidants ebselen and Tempol [31], [32] inhibit U46619-induced contraction, SrcFK auto-phosphorylation (Tyr416) and phosphorylation of MYPT-1 (Thr850) and MLC_20_ (Ser19). These experiments confirm the importance of endogenous ROS in U46619-induced SrcFK activity, Rho-kinase activity and contraction in IPA. Neither antioxidant nor combined SOD/catalase had any effect on basal phosphorylation of SrcFK, MYPT-1 or MLC_20_, suggesting that only agonist-induced ROS, not basal ROS, contributes to the activation of SrcFK and Rho-kinase. U46619-induced contraction and all three phosphorylation responses were also inhibited by the selective SrcFK antagonist PP2 [45], thus confirming previous reports that SrcFK are acting upstream of Rho-kinase in this model [24].

The fact that individually, Tempol, ebselen and PP2 all completely prevent U46619-induced phosphorylation responses suggests that ROS and SrcFK may be acting as part of the same signalling pathway, resulting in activation of Rho-kinase and MLCK. To test this assumption and to examine the relative contribution of superoxide and H_2_O_2_ to this signalling pathway, we also examined the effects of exogenous ROS on contraction and phosphorylation responses. LY83583 is a quinolinequinone that acts as a substrate for the enzyme quinone oxidoreductase, thus enhancing cytosolic superoxide generation [33]. We previously showed that low dose LY83583 greatly enhances PGF_2α_-induced Rho-kinase activity and contraction in IPA [15]. In the present study, we demonstrated a similar enhancement of a U46619-induced pre-constriction by LY83583 and that this enhancement was dependent on both ROS (Tempol- and ebselen-sensitive) and SrcFK (PP2-sensitive). Furthermore, LY83583 induced PP2-sensitive SrcFK auto-phosphorylation and tyrosine phosphorylation of multiple proteins in IPA. We also showed that exogenously applied H_2_O_2_ induced PP2-sensitive contraction and SrcFK auto-phosphorylation in IPA. Therefore, both superoxide and H_2_O_2_ are capable of activating SrcFK. We previously showed that while 30 µM H_2_O_2_ raises intracellular [Ca^2+^] in PASMC, it does not influence MYPT-1 phosphorylation [16] and does not induce Rho-kinase translocation, while LY83583 does both [15]. Furthermore, the LY83583-induced translocation was prevented by SOD, suggesting that ROS-induced activation of Rho-kinase in IPA is superoxide-specific. However, in the present study, we found that contraction and phosphorylation responses to U46619 were partially inhibited by catalase and further inhibited by combined SOD/catalase, suggesting both superoxide and H_2_O_2_ contribute to this kinase activity and contraction in response to GPCR stimulation. One potential implication of this result is that *endogenously* (intracellular) generated H_2_O_2_ has different actions to that applied exogenously. This may be related to the normal compartmentalisation of cellular ROS generators, ROS effectors and cellular antioxidant systems being disrupted by the application of exogenous H_2_O_2_ [6], [46].

Having established that U46619-induced SrcFK activity is ROS-dependent, we sought to determine whether this occurred via direct oxidation of c-Src. Our cysteine oxidation data suggest that at least a component of the ROS-dependent activation of c-Src occurs via this route. The appearance of additional c-Src bands following pegylation of oxidised cysteine residues, particularly one approximately 5 kDa larger than the main band, suggests oxidation of a single cysteine residue on c-Src [29]. Intensification of this band by U46619, LY83583 and H_2_O_2_ and prevention of this intensification by Tempol, suggest that both exogenous and endogenous ROS are oxidising a fraction of cellular c-Src in IPA. Intensity of the main non-pegylated c-Src band was not significantly reduced by any of the treatments, suggesting that most of the cellular c-Src remains in the non-oxidised state. It may be that the relatively small fraction of total cellular c-Src that is being oxidised under these conditions fully accounts for the subsequently observed ROS-dependent downstream signalling events, since broadly similar order of magnitude increases in c-Src oxidation and SrcFK auto-phosphorylation were recorded in response to U46619 and LY83583. There is previous evidence in other tissues for activation of c-Src by direct cysteine oxidation, particularly Cys487 and Cys235, which when oxidised, trigger auto-phosphorylation [47]. Alternatively, there may also be indirect activation of SrcFK through oxidative inhibition of negative regulators of SrcFK, such as c-Src kinase or specific tyrosine phosphatases [48], [49]. Taken together, the results so far suggest a sequence of events starting with ROS production, followed by ROS-dependent activation of SrcFK, and subsequent activation of Rho-kinase and contraction.

SrcFK and Rho-kinase are also both activated by hypoxia in pulmonary artery and are major contributors to hypoxic pulmonary vasoconstriction (HPV), and as with G-protein coupled receptor signalling, we have shown that SrcFK is acting upstream of Rho-kinase in response to hypoxia [11]. However, in that previous study we did not establish the role of endogenous ROS in Rho-kinase activation. There is strong evidence that HPV is initiated by increased generation of ROS from complex III of the mitochondrial ETC, and HPV has been shown to be abolished by inhibitors of complex I and III such as rotenone and myxothiazol, as well as by antioxidants, including ebselen and Tempol [9], [50], [51], also reviewed in [10]. The data reported here complements such reports by showing that myxothiazol and rotenone inhibited hypoxia-induced SrcFK auto-phosphorylation and, as predicted by the ROS-sensitivity of Rho-kinase, they also inhibited MYPT-1 and MLC_20_ phosphorylation induced by hypoxia. The relative selectivity of rotenone and myxothiazol for mitochondrial over GPCR-induced ROS are confirmed by the lack of significant effect of these inhibitors on U46619-induced SrcFK/MYPT1/MLC_20_ phosphorylation. Together these results are consistent with the concept that SrcFK are activated by mitochondrial ROS and mediate the subsequent activation of Rho-kinase during HPV. Notably, as rotenone and myxothiazol block HPV [9], but did not significantly affect U46619-induced contraction (this study), mitochondria are unlikely to be the source of the U46619-induced elevation of ROS, which as discussed above, is more probably NADPH oxidase [1], [2], [9].

### 4.2 Interaction between ROS, SrcFK, AHRGEF1 and RhoA

Having established that SrcFK contribute to ROS-mediated Rho-kinase activation in response to U46619 stimulation and hypoxia, we attempted to further clarify the link between SrcFK and Rho-kinase by identifying molecular targets/binding partners for SrcFK. Rho-kinase requires prior activation of the monomeric G-protein RhoA [52], and while neither RhoA nor Rho-kinase are known to require direct tyrosine phosphorylation, several members of the Rho guanine nucleotide exchange factor (RhoGEF) family of proteins which activate RhoA by promoting the exchange of bound GDP for bound GTP, have been shown to be activated by direct tyrosine phosphorylation [26], [53], [54], [55], in addition to *or instead of* interaction with Gα_12/13_ [19], [56]. These include the RGS domain-containing GEFs ARHGEF1 (p115-RhoGEF), ARHGEF11 (PDZ-RhoGEF) and ARHGEF12 (LARG), amongst others. Inhibition of U46619 or LY83583 induced contraction by Y16, a selective inhibitor of RhoA activation by RGS-domain containing RhoGEFs [38], supports the importance of these proteins.

We specifically chose to examine ARHGEF1 in more detail because it has previously been implicated in AngII-induced hypertension and shown to be tyrosine phosphorylated by Janus kinase-2 (JAK2) in mesenteric artery as an essential step in its activation by AngII [26]. In live PASMC we examined translocation of EmGFP-tagged RhoA and EmGFP-tagged ARHGEF1, during stimulation by U46619 or LY83583. We found that both stimuli triggered reversible and reproducible translocation of both proteins to distinct spots or patches on the cellular periphery, and that these responses were dependent on both SrcFK activity (PP2-inhibitable) and ROS (Tempol or ebselen-inhibitable). Although further work would be required to identify the cellular structures to which the two proteins translocate, previous studies suggest they may be integrin-associated focal attachments [57], [58]. Assuming that this translocation does indeed precede or is concomitant with ARHGEF1 and RhoA activation [36], [56], our results suggest that ROS and SrcFK are likely to be stimulating Rho-kinase via prior activation of ARHGEF1, followed by activation of RhoA. Direct activation of RhoA by ROS independently of RhoGEF binding has been described previously [18], but this is unlikely to be contributing to our results because RhoA translocation induced by either U46619 or LY83583 was nearly abolished by co-transfection with an ARHGEF1 siRNA, confirming that ROS-dependent and SrcFK-dependent activation of ARHGEF1 is likely to be an essential step in the activation of RhoA and therefore of Rho-kinase. While U46619 will be inducing translocation via a G-protein coupled receptor, subsequent NOX-derived ROS production and SrcFK activity, LY83583 will be doing so via ROS and SrcFK, but independently of a G-protein coupled receptor. Thus, although ARHGEF1 is previously known to require direct activated by G_12/13_ [19], [56], our results suggest that ARHGEF1 may be activatable by any stimuli that induce ROS production and or SrcFK-dependent tyrosine kinase activity.

Our observation that co-immunoprecipitation of ARHGEF1 and c-Src in IPA is enhanced by U46619 suggests that a close association between the two proteins may occur. Furthermore, the co-immunoprecipitation was prevented by Tempol, suggesting that the association is ROS-dependent. This is also supported by the observation that LY83583 (this study) and PGF_2α_ [24] both enhance SrcFK-dependent tyrosine phosphorylation of multiple proteins in IPA, including one at approximately 115 kDa, the expected location of ARHGEF1. The co-immunoprecipitation of c-Src and ARHGEF1 was recorded after 10 min exposure to U46619, so the complex thus formed is presumably quite stable. Further studies would be required to determine whether this indicates direct phosphorylation of ARHGEF1 by c-Src. Although there is a precedent for the requirement of such phosphorylation in ARHGEF1 activation, albeit by JAK2 [26], there is as yet no direct evidence that members of the Src family of kinases themselves phosphorylate any RhoGEF other than the oncogenic Vav or Tiam1, despite being implicated in activation of both RhoA and Rac-1 [59], [60]. SrcFK are, however, known to phosphorylate and activate other non-receptor tyrosine kinases such as focal adhesion kinase, PYK2 and JAK2, in response to multiple stimuli, including G-protein coupled receptors and exogenous ROS [61], [62], [63], [64], [65], while all three of the aforementioned kinases have been shown to phosphorylate RhoGEFs [26], [66], [67]. It is reasonable to suggest, therefore, that if not directly, SrcFK may be mediating the activation of ARHGEF1 and RhoA via one or more of these other kinases.

## 5 Conclusion

**Fig. 9:**
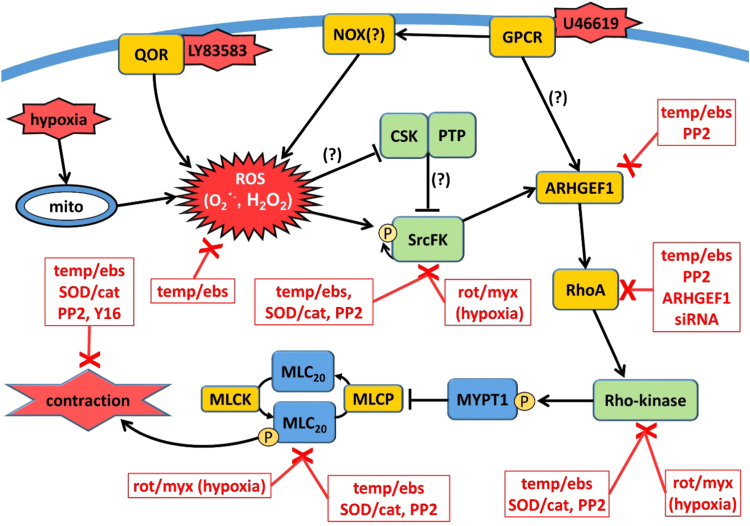
**Graphical Summary. ROS-induced contractile signalling pathway in rat pulmonary artery.** Reactive oxygen species (ROS: superoxide and H_2_O_2_) generated in response to GPCR (presumably via NADPH-oxidase, NOX), LY83583 (via quinone oxidoreductase, QOR), or hypoxia (from mitochondria, mito), activate Src-family kinases (SrcFK) either via direct oxidation or via oxidative inhibition of c-Src kinase (CSK) or inhibitory tyrosine phosphatases (PTP). This is followed by sequential activation of ARHGEF1, RhoA and Rho-kinase, resulting in enhanced MYPT-1 and MLC_20_ phosphorylation and contraction. Boxed red text indicates treatments/antagonists that inhibit responses or activity of each component in the pathway. Temp/ebs = antioxidants Tempol & ebselen; SOD/cat = superoxide dismutase and catalase; PP2 = SrcFK inhibitor; rot/myx = mitochondrial electron transfer chain inhibitors rotenone & myxothiazol; Y16 = inhibitor of RGS domain containing RhoAGEFs. Question mark indicates steps shown previously but not examined in this study.

Our study characterises a contractile signalling pathway in pulmonary artery that incorporates ROS, SrcFK, ARHGEF1, RhoA and Rho-kinase. As outlined in the graphical summary (Fig. 9), we identify SrcFK as key proximal ROS effectors in response to both GPCR activation and hypoxia. We also propose ARHGEF1 as a possible target for ROS-dependent SrcFK activity upstream of RhoA/Rho-kinase activation in IPA contractile responses. Further investigation into the interaction between these proteins downstream of ROS production, particularly SrcFK, ARHGEF1 and Rho-kinase, may inform the identification of new therapeutic targets for the treatment of cardiovascular diseases associated with oxidative stress, such as pulmonary hypertension.

## Funding

This work was supported by British Heart Foundation [grant number FS/12/43/29608 to G.K.] and Wellcome Trust [grant #087776 to J.P.W].

## Conflict of interest

None.
