# Supplementary material for: ROS-dependent activation of RhoA/Rho-kinase in pulmonary artery: Role of Src-family kinases and ARHGEF1
Source: Free Radic Biol Med. 2017 Sep;110:316–31. doi: 10.1016/j.freeradbiomed.2017.06.022 (PMC5542024; doi:10.1016/j.freeradbiomed.2017.06.022)
Supplement: Supplementary file 1 — Supplementary material [file mmc1.docx]

**
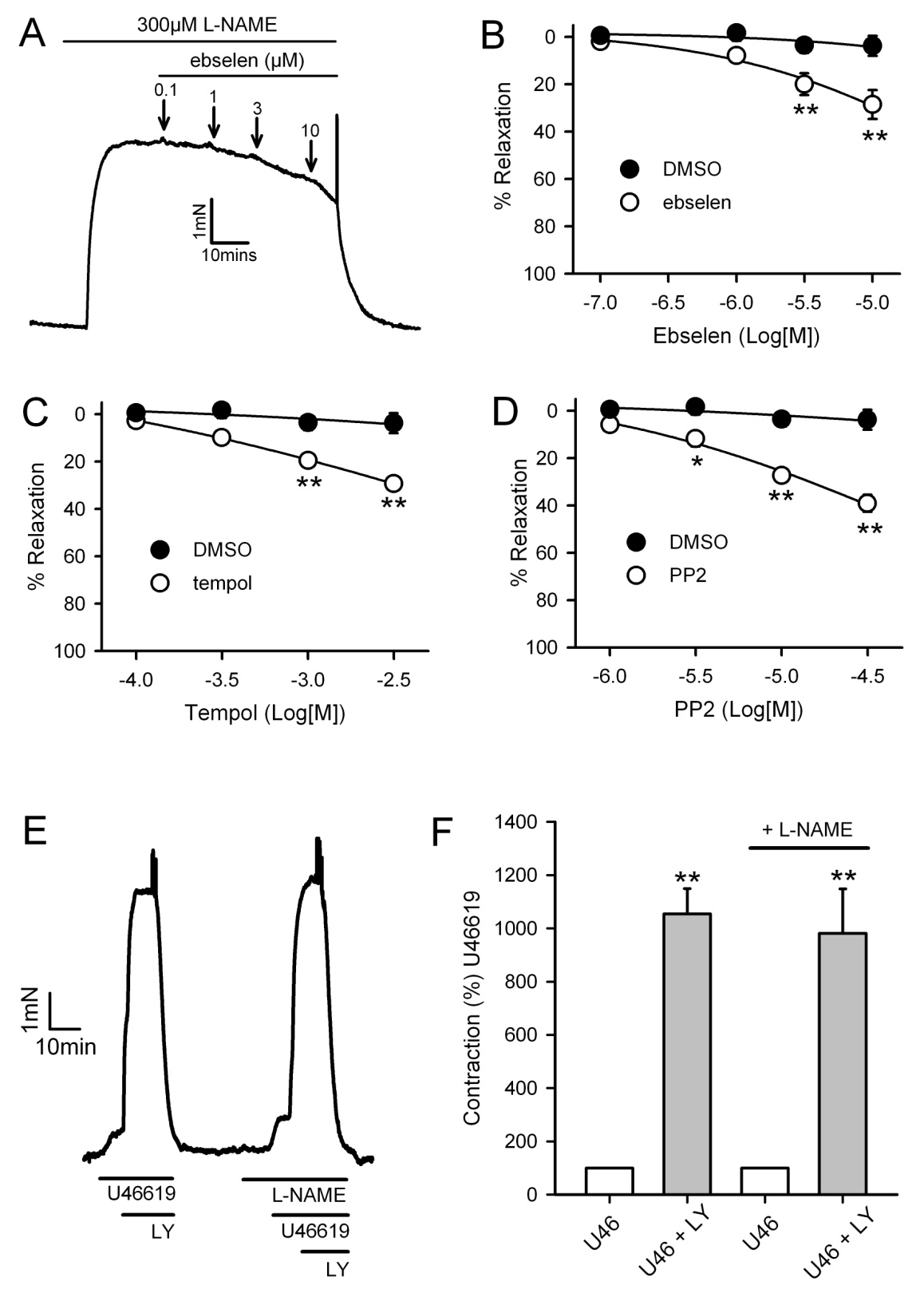
Figure S1. L-NAME does not alter the effects of antioxidants or LY83583 on U46619-induced contraction**

**A-D**: Representative trace (A) and mean data for the effects of L-NAME (300µM) on concentration-dependent relaxation responses to ebselen (B, n=6), Tempol (C, n=6) and PP2 (D, n=7), plotted against DMSO vehicle control (n=9), in IPA pre-constricted with 100nM U46619. In A, arrows indicate where each dose of ebselen was added. *P<0.05, **P<0.01 vs. DMSO control (2-way ANOVA). **E, F:** Effect of L-NAME (300µM) on 1µM LY83583-induced enhancement of U46619-mediated contraction in IPA. U46619 concentration (< 2nM) was adjusted to ensure initial contraction was ~10% of KPSS in amplitude. n= 6, **P<0.01 vs. U46619 alone, paired t-test.

**
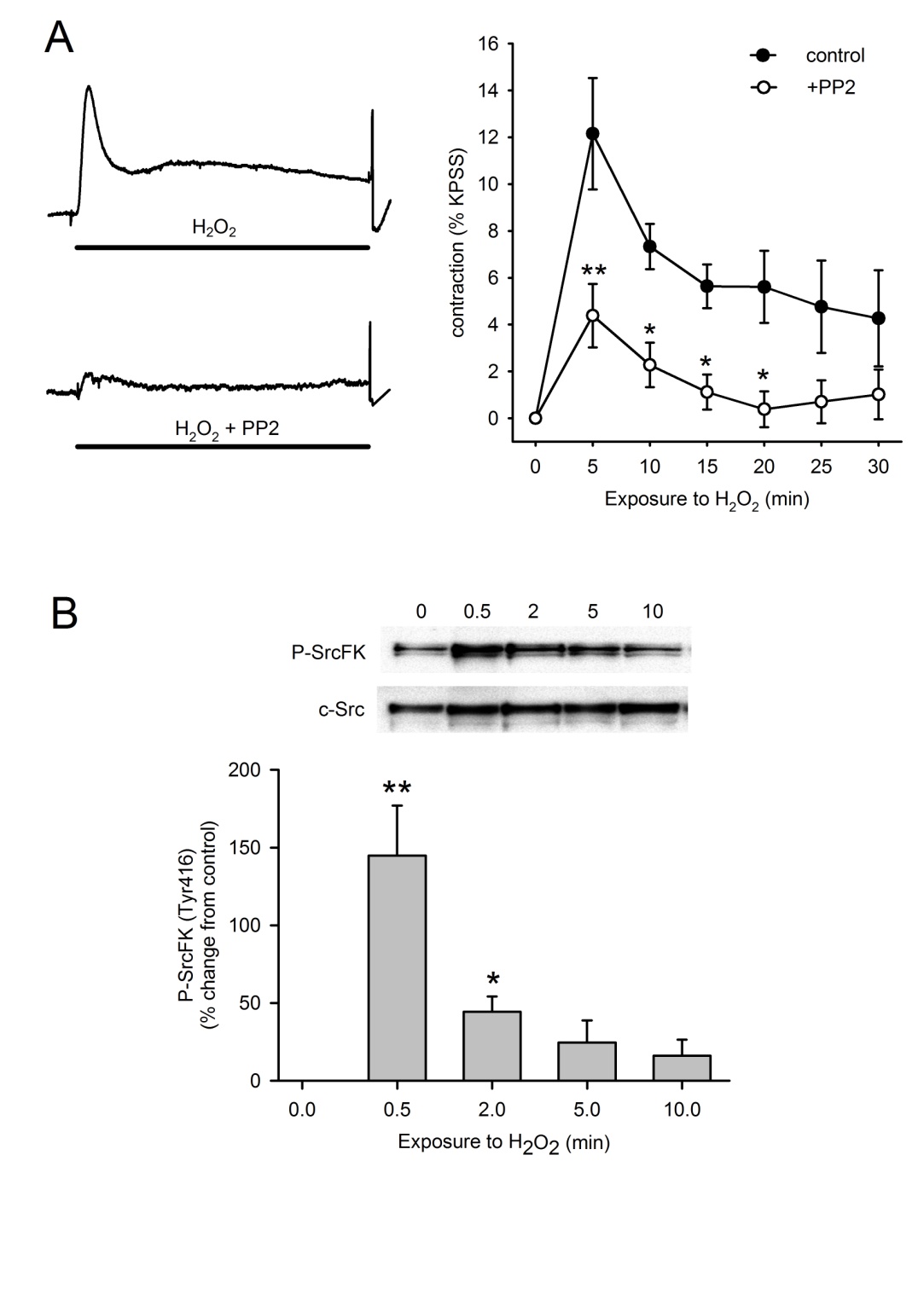
**

**Supplementary figure S2. A:** 30 min exposure to 30µM H_2_O_2_ induces contraction in IPA which is partially suppressed by the SrcFK antagonist PP2 (30µM, n= 7-8, *P<0.05, **P<0.01 vs. control). Comparison by 2-way RM-ANOVA. **B:** H_2_O_2_ (30µM) time-dependently enhances SrcFK auto-phosphorylation (Tyr416) in IPA (n= 4-9, #P<0.05 vs. control, ##P<0.01 vs. control). Comparison by 1-way ANOVA.

**
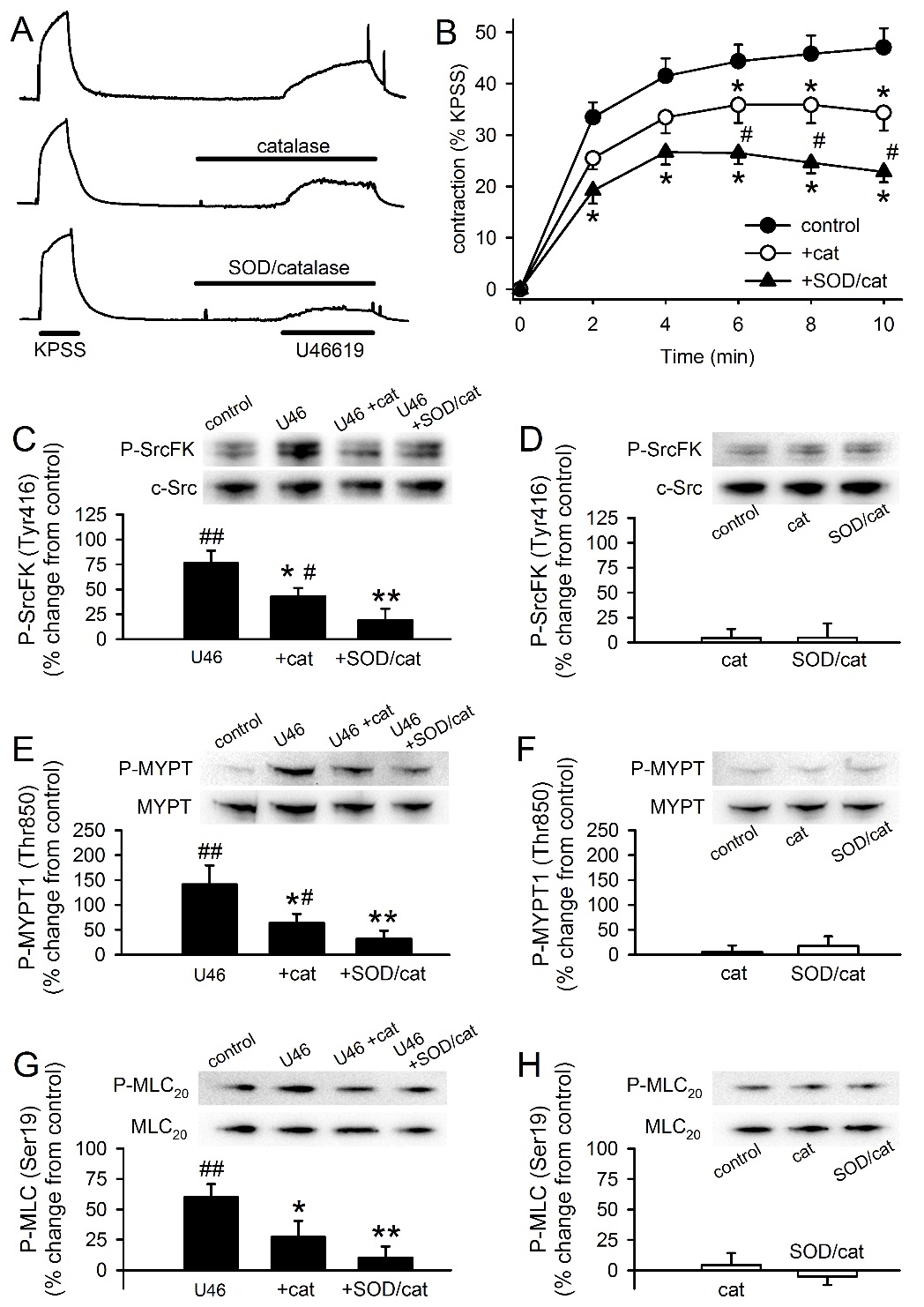
**

**Supplemental Figure S3. Effects of catalase and superoxide dismutase on contraction and on SrcFK, MYPT-1 and MLC_20_ phosphorylation induced by U46619 in IPA.**

**A, B:** Contraction induced by U46619 (100nM) is partially suppressed by catalase (200 U/ml, 10 min pre-incubation, *P<0.05 vs. control), but further suppressed by the combined action of catalase and superoxide dismutase (SOD, 200 U/ml, 10 min pre-incubation, *P<0.05 vs. control, #P<0.05 vs. catalase only). n=10 for all treatments. Comparisons by 2-way RM-ANOVA. **C, E, G:** SrcFK auto-phosphorylation (Tyr416, **C**), and phosphorylation of MYPT-1 (Thr850, **E**) and MLC_20_ (Ser19, **G**), is enhanced by U46619 in IPA (U46, 100nM, 30 min, ##P<0.01 vs. control). This enhancement is partially suppressed by catalase (200 U/ml, 10 min pre-incubation, *P<0.05 vs. U46 only, #P<0.05 vs. control), but further suppressed by the combined action of catalase and superoxide dismutase (SOD, 200 U/ml, 10 min pre-incubation, **P<0.01 vs. U46 only). **D, F, H:** Basal SrcFK auto-phosphorylation (**D**) and basal phosphorylation of MYPT-1 (**F**) or MLC_20_ (**H**) were all unaffected by catalase or combined SOD/catalase. n=10-12. Comparisons by 2-way ANOVA.

**
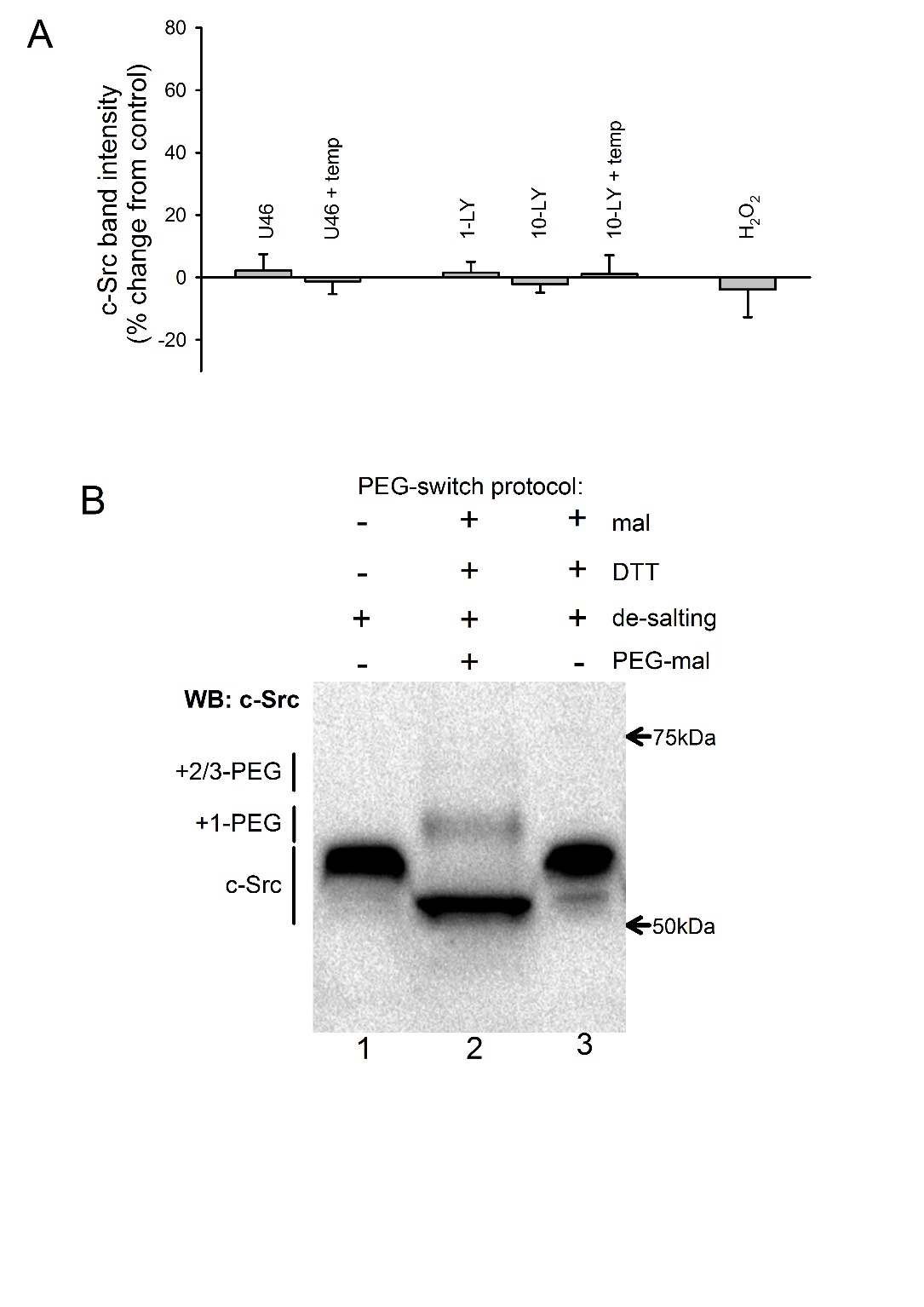
**

**Supplementary figure S4. PEG-switch cysteine oxidation assay. A:** When subjected to the PEG-switch cysteine oxidation assay, prior treatement of IPA with U46619 (U46, 100nM, 30 min, n= 9), LY83583 (LY, 1 or 10µM, 30 min, n=7-8) and H_2_O_2_ (30µM, 30 min, n=5) did not significantly alter the intensity of the main non-pegylated c-Src band. Co-treatment with Tempol was similarly without significant effect (temp, 3mM, n= 6-8). Comparisons by 1-way ANOVA. **B:** Control experiments verifying that additional bands are specifically due to pegylation. **1**: The de-salting step alone does not alter the appearance of the single strong c-Src band seen in normal WB (eg Fig 3). **2**: The full PEG-switch protocol accelerates gel migration, but more importantly induces the appearance of additional bands, particularly one approximately 5 kDa above the main band, indicating pegylation of c-Src on at least one oxidized cysteine residue. **3**: Removing the final PEG-maleimide (PEG-mal) step prevents pegylation, resulting in a single strong band being detected, identical to that seen in lane 1 and in normal WB. All samples shown here were treated with 100nM U46619 for 30 min prior to application of the PEG-switch protocol.


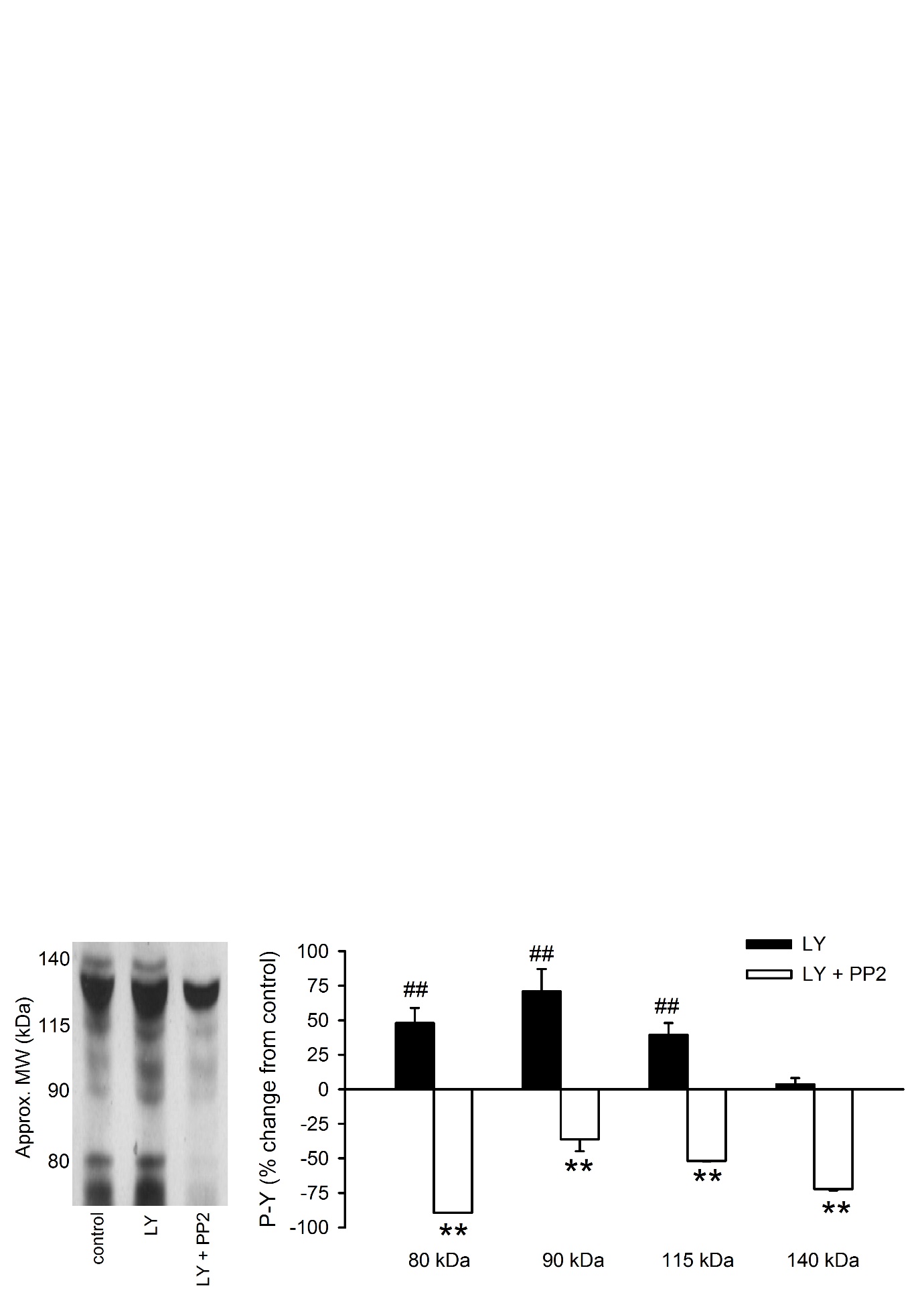


**Supplementary figure S5. SrcFK-dependent ROS-induced tyrosine phosphorylation in IPA.** Treatment with LY83583 (LY, 1µM, 30 min) enhanced tyrosine phosphorylation of multiple proteins, including bands of approximately 80, 90, 115 and 140 kDa. These phosphorylations were considerably sensitive to SrcFK inhibition with PP2 (30µM, n=4). ##P<0.01 vs. control, *P<0.05, **P<0.001 vs. LY alone (2-way ANOVA).


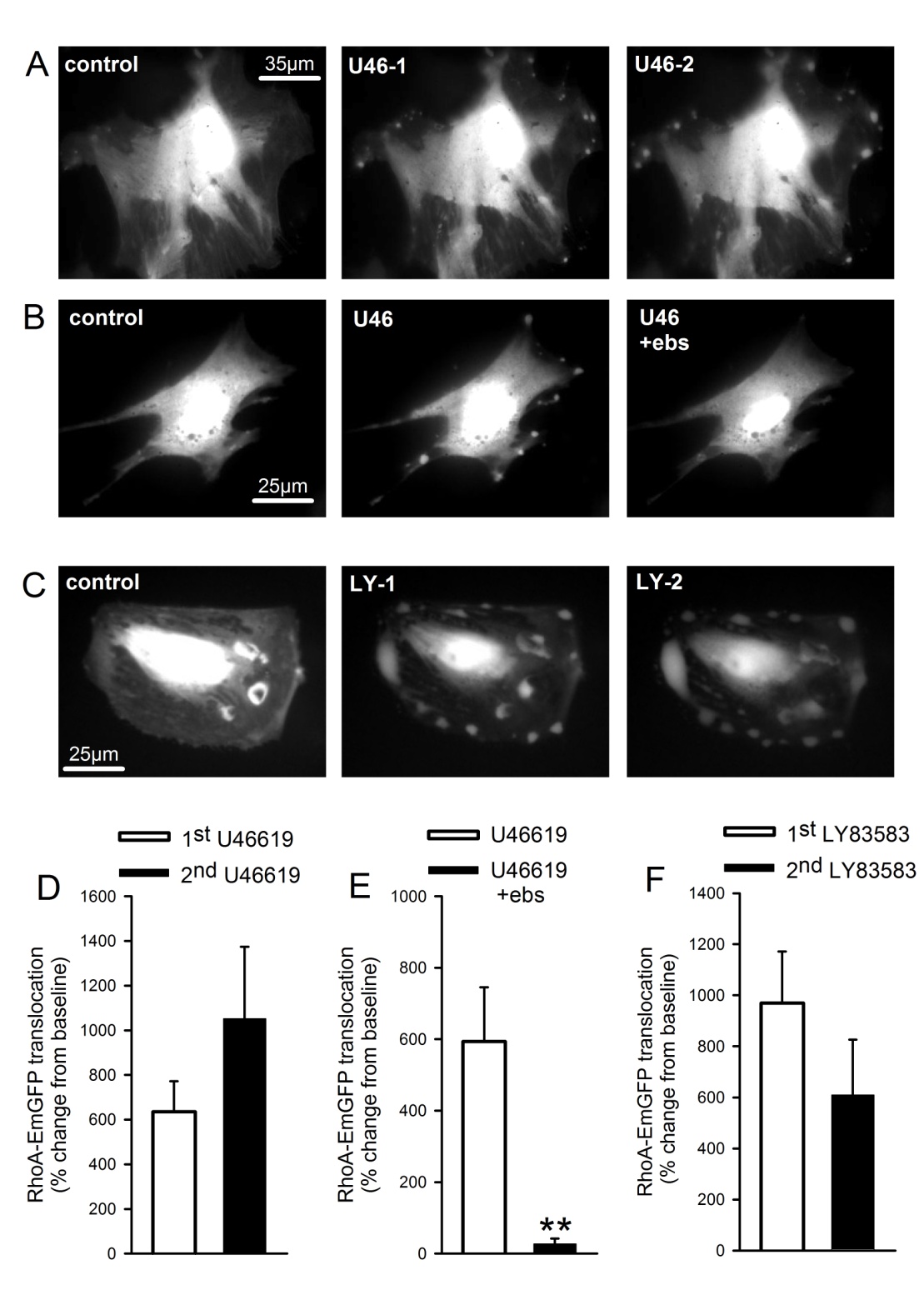
**Supplementary figure S6. Control responses for effects of U46619 and LY83583 on RhoA-EmGFP translocation in PASMC.**

**A-C:** Fluorescent imaging of representative RhoA-EmGFP transfected PASMC at rest (left panels), in the presence of stimulus (middle panels **A-B**: U46619, U46, 100nM or **C**: LY83583, LY, 1µM) or after washout followed by repeated exposure to stimulus in the presence of either vehicle (DMSO), or ebselen (right panels). In the absence of inhibitor, fluorescence concentrates into spots or patches on the cell periphery during stimulus and this is repeated after washout and reapplication of stimulus. **D-F:** Quantification of spot/patch fluorescence intensity expressed as % change above control levels during stimulus ± inhibition (**D**-**E**: U46619, **F**: LY83583). **P<0.001 vs. U46619, 2-way ANOVA, n=7-10 cells from a total of seven different cell lines. Each measurement is combined from at least 3 spots/patches from each cell.

**
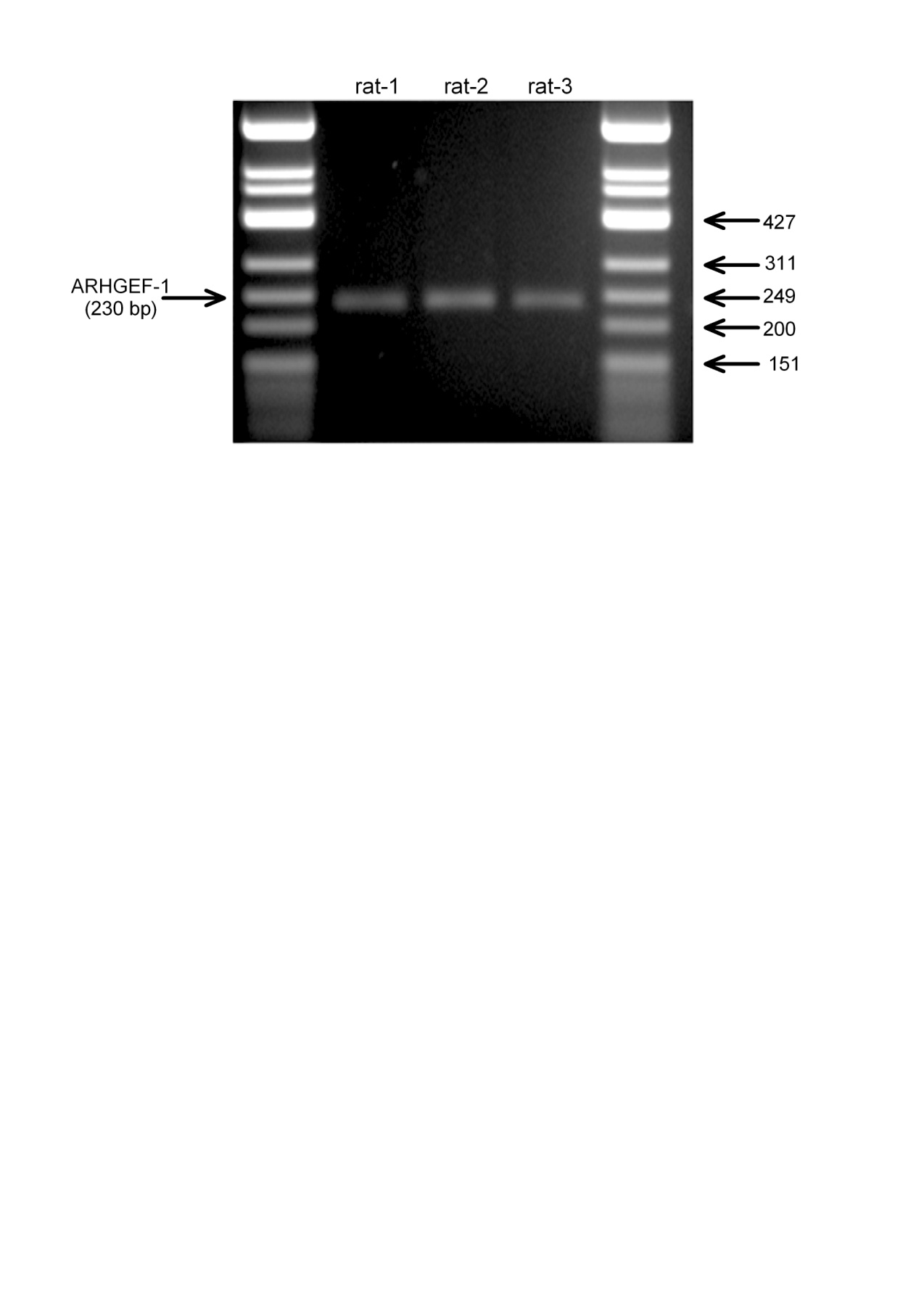
**

**Supplementary Figure S7. ARHGEF1 expression in IPA**

RNA was isolated from IPA of three different rats and subjected to reverse transcription as previously described ^1^. RT-PCR primer pair for rat ARHGEF1 were designed using Primer3web version 4.0 (http://primer3. ut.ee) and synthesized by Sigma-Aldrich (St Louis, MO, USA). ARHGEF1 (accession no. BC091218): sense GTGCTATTGCTGGACGACCT, antisense GTCTGTGCCACCAGCTCATA. PCR was carried out using PuReTaq Ready-To-Go PCR Beads (GE Healthcare, Piscataway, NJ, USA) and the PCR products (reaction equivalent on 20 ng reverse transcribed RNA) were analysed by electrophoresis on 2.8% agarose gels run in TAE buffer (National Diagnostics, Hessle, UK) with PhiX174 DNA/HinfI Marker (Thermoscientific Inc., Waltham, MA, USA). Product after 32 cycles appears at expected amplifier size of 230 bp.

1. Knock GA, Snetkov VA, Shaifta Y, Drndarski S, Ward JP, Aaronson PI. Role of src-family kinases in hypoxic vasoconstriction of rat pulmonary artery. Cardiovasc Res 2008;80:p 453-462.


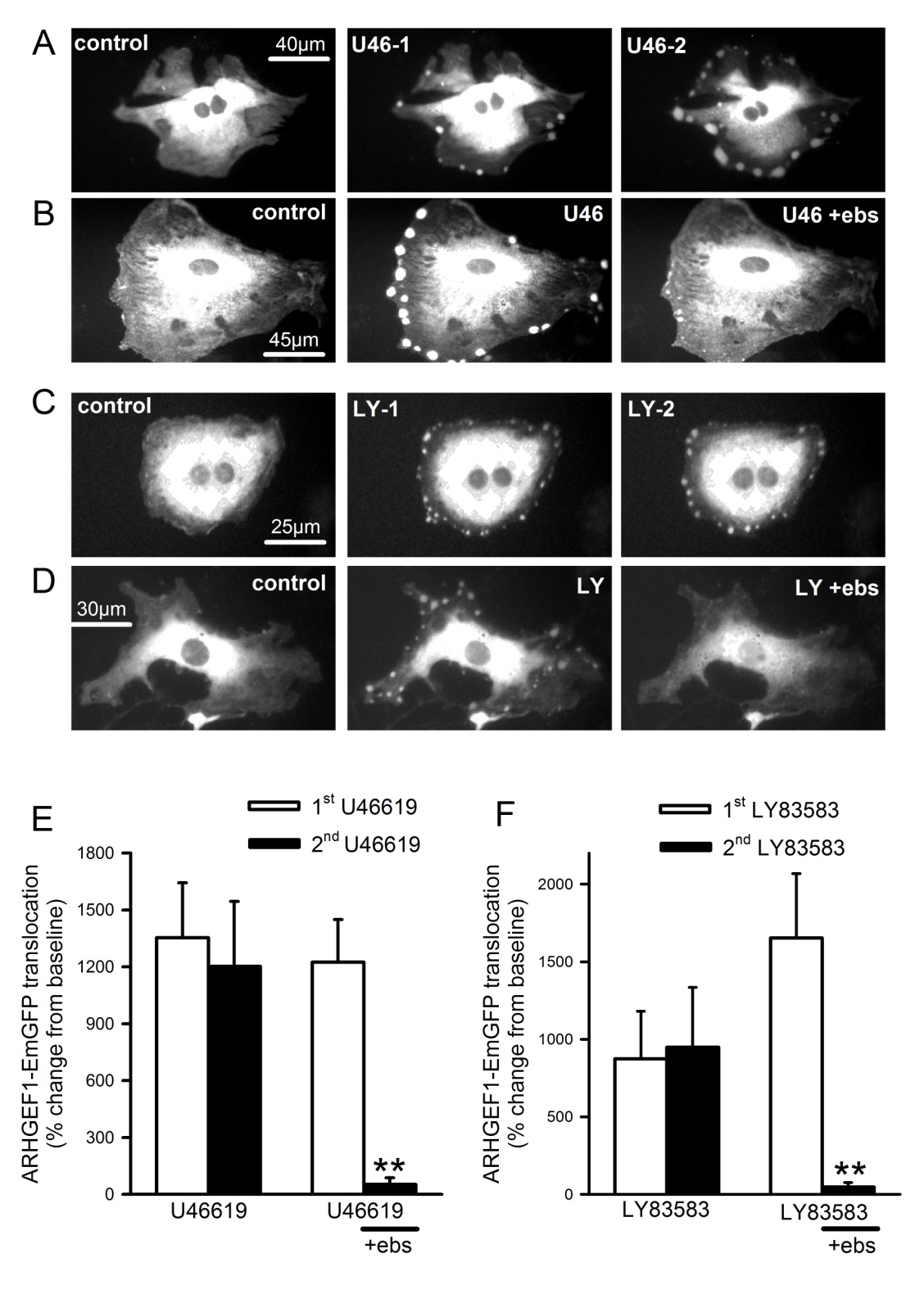
**Supplementary figure S8.** **Control responses for effects of U46619 and LY83583 on ARHGEF1-EmGFP translocation in PASMC.**

**A-D:** Fluorescent imaging of representative ARHGEF1-EmGFP transfected PASMC at rest (left panels), in the presence of stimulus (middle panels **A-B**: U46619, U46, 100nM or **C-D**: LY83583, LY, 1µM) or after washout followed by repeated exposure to stimulus in the presence of either vehicle (DMSO), or ebselen (right panels). In the absence of inhibitor, fluorescence concentrates into spots or patches on the cell periphery during stimulus and this is repeated after washout and reapplication of stimulus. **E-F:** Quantification of spot/patch fluorescence intensity expressed as % change above control levels during stimulus ± inhibition (**E**: U46619, **F**: LY83583). **P<0.001 vs. U46619, 2-way ANOVA, n=6-9 cells from a total of seven different cell lines. Each measurement is combined from at least 3 spots/patches from each cell.


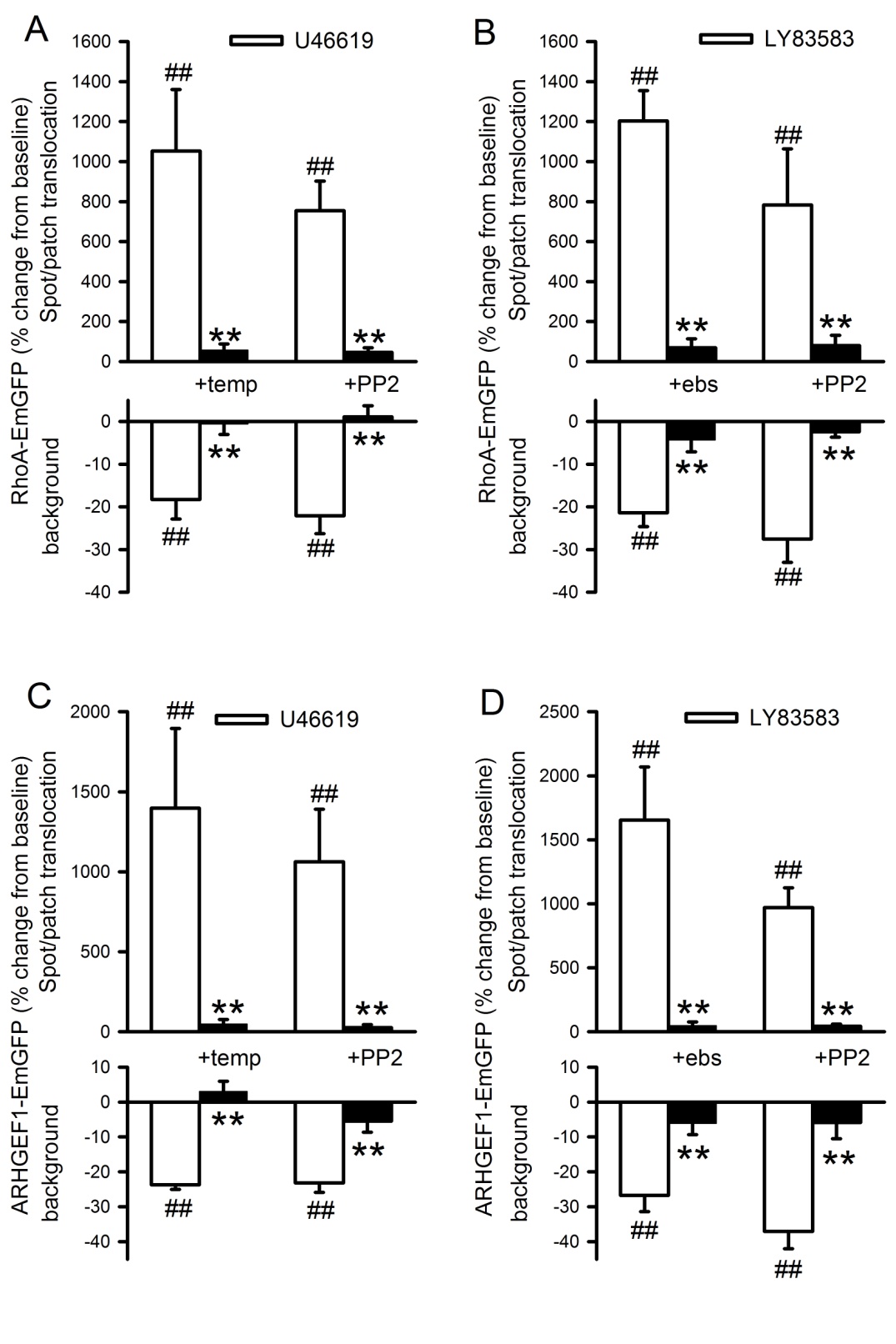


**Supplementary figure S9. RhoA-EmGFP and ARHGEF1-EmGFP translocation is accompanied by reductions in cytosolic background fluorescence during U46619 and LY83583 stimulation.**

Quantification of spot/patch fluorescence intensity (upper panels) and background cytosolic fluorescence (lower panels) during stimulus ± inhibition, expressed as % change above or below baseline levels (**A:** RhoA-EmGFP, U46619-stimulated, **B:** RhoA-EmGFP, LY83583-stimulated, **C**: ARHGEF1-EmGFP, U46619-stimulated, **D**: ARHGEF1-EmGFP, LY83583-stimulated). Appearance of bright spots/patches is accompanied by darkening of nearby background, and both responses are similarly inhibited by Tempol (temp), ebselen (ebs) and PP2. ##P<0.001 vs. baseline, **P<0.001 vs. U46619, 2-way ANOVA, n=6-9 cells from a total of seven different cell lines. Each measurement is combined from at least 3 regions from each cell.
